# Maximin Efficiencies under Treatment-Dependent Costs and Outcome Variances for Parallel, AA/BB, and AB/BA Designs

**DOI:** 10.1155/2018/8025827

**Published:** 2018-10-01

**Authors:** Math J. J. M. Candel

**Affiliations:** Department Methodology and Statistics, CAPHRI Care and Public Health Research Institute, Maastricht University, Maastricht, Netherlands

## Abstract

If there are no carryover effects, AB/BA crossover designs are more efficient than parallel (A/B) and extended parallel (AA/BB) group designs. This study extends these results in that (a) optimal instead of equal treatment allocation is examined, (b) allowance for treatment-dependent outcome variances is made, and (c) next to treatment effects, also treatment by period interaction effects are examined. Starting from a linear mixed model analysis, the optimal allocation requires knowledge on intraclass correlations in A and B, which typically is rather vague. To solve this, maximin versions of the designs are derived, which guarantee a power level across plausible ranges of the intraclass correlations at the lowest research costs. For the *treatment effect*, an extensive numerical evaluation shows that if the treatment costs of A and B are equal, or if the sum of the costs of one treatment and measurement per person is less than the remaining subject-specific costs (e.g., recruitment costs), the maximin crossover design is most efficient for ranges of intraclass correlations starting at 0.15 or higher. For other cost scenarios, the maximin parallel or extended parallel design can also become most efficient. For the *treatment by period interaction*, the maximin AA/BB design can be proven to be the most efficient. A simulation study supports these asymptotic results for small samples.

## 1. Introduction

The standard design of a randomized clinical trial is the parallel group design: subjects are randomly assigned to one of two treatments, say A or B. An alternative, well-known design is the AB/BA crossover trial in which subjects receive both treatments, A and B, but the sequencing of the treatments is opposite for two randomly allocated groups [1, 2]. An AB/BA crossover trial is considered most suited when examining treatments for chronic or ongoing diseases, such as rheumatism, chronic obstructive pulmonary disease, or (frequent) heartburn. In these cases, there is no real possibility that the disease gets cured, and the aim is to moderate the effects of the disease [2]. A third design that we will consider involves treatment sequences AA and BB. This design extends the parallel design across two treatment periods, allows for testing treatment by time interaction effects, and is a realistic alternative for the AB/BA design in case the treatment regime should continue.

If the outcome variable is continuous and (approximately) normally distributed, the data can be analyzed by mixed effects regression [3]. Of primary interest is testing the treatment effect of, for instance, a new medication for chronic obstructive pulmonary disease. A relevant issue then is which design is the most efficient in estimating the treatment effect, thereby yielding maximum power for testing this effect. Such optimality has already been examined when comparing crossover and parallel designs [2] and when comparing all three designs introduced before [4, 5]. If there are no carryover effects and no dropouts, the sample sizes are equal and equally allocated to the treatments, an AB/BA design yields more efficient estimates of the treatment effect than a parallel and extended parallel design and consequently, will yield more power to test this effect.

The present study extends results on the relative efficiencies of these designs in that (a) optimal instead of equal treatment allocation is examined, (b) allowance is made for treatment-dependent outcome variances, and (c) next to treatment effects, also treatment by period interaction effects are examined. Outcome variances may differ between treatments [6, 7]. This also is to be expected if treatments differ in terms of their effectiveness. Furthermore, since research costs and outcome variances may differ between treatments, equal allocation to treatments may not be the most efficient. The issue then is how to allocate subjects to treatments such that a design's efficiency is optimized, and how different designs relate in terms of efficiency under such optimal allocation. Optimal allocation requires a priori knowledge on parameters of the analysis model, that is, intraclass correlations for the mixed effects model that we consider. Since this knowledge typically is rather vague, optimal allocations and corresponding efficiencies for maximin versions of the (extended) parallel design and crossover design will be derived. These maximin designs guarantee a power level across plausible ranges of the intraclass correlations at the lowest research costs.

In designs where treatments are successively given to the same group of subjects, carryover may occur. For the AB/BA trial, it may be that, in the AB sequence treatment, A still has an effect on the outcome, when B has been given and the second measurement is done. When in the BA sequence, the effect of B is present, once A has been administered and this effect differs from the carryover effect for the AB sequence, differential carryover occurs. The present study assumes that differential carryover can be safely excluded or is negligible and that this effect does not need to be estimated in analyzing the data.

The paper is structured as follows.  Section 2 will present the linear mixed model for analyzing data from each of the three designs.  Section 3 will introduce the efficiency criterion and will provide asymptotic expressions for this criterion in the case of maximum likelihood estimation of the treatment effect. Starting from a flexible cost function, optimal allocations to treatments will be derived as well as resulting design efficiencies. Since the efficiencies depend on the intraclass correlations and knowledge on these parameters is often limited, in Section 4, we will derive maximin designs.  Section 5 will show to what extent the asymptotic efficiencies translate into desired power levels for small sample sizes.  Section 6 will give an application of the results, and Section 7 will discuss some issues for further research.

## 2. Linear Mixed Effects Models

In the case of a parallel design, an extended parallel design, and a crossover design, the subjects are randomly allocated to one of the two arms. In a parallel design to treatment A or treatment B, in an extended parallel design, they are allocated to treatment sequence AA or BB, and in a crossover trial to treatment sequence AB or BA. We consider a quantitative outcome variable, denoted as *y*_*ij*_ for person *j* (*j*=1,…, *N*) at measurement occasion *i*, and assume *y*_*ij*_ is (approximately) normally distributed.

For a parallel design and outcome variances that differ between treatments A and B, simple linear regression with heterogeneous variances may be an adequate tool for data analysis:

(1) $$\begin{array}{c} y_{ij}=\beta_{0}+\beta_{1}{\text{treat}}_{ij}+\delta_{ij}^{\text{A}}\left(1-{\text{treat}}_{ij}\right)+\delta_{ij}^{\text{B}}{\text{treat}}_{ij}, \end{array}$$

where treatment is coded 0 for persons having treatment A and coded 1 for persons having treatment B, and *δ*_*ij*_^A^ and *δ*_*ij*_^B^ are normally distributed, with mean 0 and variances *σ*_A_^2^ and *σ*_B_^2^, respectively. The random terms *δ*_*ij*_^A^ and *δ*_*ij*_^B^ can be thought of as consisting of a random person (between-subject) effect, *u*_0*j*_, and a treatment-dependent random error (within-subject) effect, *ε*_*ij*_^A^ and *ε*_*ij*_^B^. In formula, *δ*_*ij*_^A^=*u*_0*j*_+*ε*_*ij*_^A^, and *δ*_*ij*_^B^=*u*_0*j*_+*ε*_*ij*_^B^. These two sources of random variation cannot be separated in a single-period parallel trial.

For a crossover AB/BA design and an extended, two-period, parallel AA/BB design, however, the variances of *u*_0*j*_ and of *ε*_*ij*_^A^ and *ε*_*ij*_^B^ can be identified. The linear regression model can then be extended with a random intercept as well as a fixed effect of time, yielding the following mixed effects model:

(2) $$\begin{array}{c} y_{ij}=\beta_{0}+\beta_{1}{\text{treat}}_{ij}+\beta_{2}{\text{time}}_{ij}+u_{oj}+\varepsilon_{ij}^{\text{A}}\left(1-{\text{treat}}_{ij}\right)+\varepsilon_{ij}^{\text{B}}{\text{treat}}_{ij}. \end{array}$$

In (2), time is coded 0 for observations at the first measurement and coded 1 for observations at the second measurement. The random terms *u*_0*j*_, *ε*_*ij*_^A^, and *ε*_*ij*_^B^ are independently normally distributed, with mean 0 and variances *σ*_0_^2^, *σ*_*ε*A_^2^, and *σ*_*ε*B_^2^, respectively. Their relation with the variances in (1) is *σ*_A_^2^=*σ*_0_^2^+*σ*_*ε*A_^2^ for treatment A and *σ*_B_^2^=*σ*_0_^2^+*σ*_*ε*B_^2^ for treatment B.

In the case we want to examine whether there is an interaction between treatment and period, the model in (2) is extended as follows:

(3) $$\begin{array}{c} y_{ij}=\beta_{0}+\beta_{1}{\text{treat}}_{ij}+\beta_{2}{\text{time}}_{ij}+\beta_{3}{\text{treat}}_{ij}\times {\text{time}}_{ij}+u_{oj}+\varepsilon_{ij}^{\text{A}}\left(1-{\text{treat}}_{ij}\right)+\varepsilon_{ij}^{\text{B}}{\text{treat}}_{ij}, \end{array}$$

where *β*_3_ represents the treatment by period interaction effect. The parameters in (1)–(3) can be estimated through maximum likelihood (ML). In what follows, we are interested in optimally estimating *β*_1_ in (1) and (2), which will be denoted as *β*_treat_, and in optimally estimating *β*_3_ in (3), which will be denoted as *β*_treat×time_. A relevant concept is the intraclass correlation, which is between-subject variation on the outcome as compared to the total outcome variation. For the models in (2) and (3),this can be expressed as *ρ*_A_=*σ*_0_^2^/(*σ*_0_^2^+*σ*_*ε*A_^2^) and *ρ*_B_=*σ*_0_^2^/(*σ*_0_^2^+*σ*_*ε*B_^2^) for treatments A and B, respectively. The larger the person (between-subject) variance as compared to the error (within-subject) variance, the larger the intraclass correlations. Note that we assume a common between-subject variance, but allow for treatment-dependent within-subject variances, leading to treatment-dependent within-subject correlations. We also define a variance ratio *ϕ*=(*σ*_0_^2^+*σ*_*ε*A_^2^)/(*σ*_0_^2^+*σ*_*ε*B_^2^)=*σ*_A_^2^/*σ*_B_^2^, which can be expressed as a ratio of the intraclass correlations, *ϕ*=*ρ*_B_/*ρ*_A_.

## 3. Optimal Allocations and Corresponding Design Efficiencies

Let $\text{Var}\left({\hat{\beta }}_{x}|\xi \right)$ denote the variance of the estimator of the treatment effect *β*_1_ in (1) or (2) or the variance of the treatment by the period interaction effect *β*_3_ in (3), given a design *ξ*. The efficiency of an estimator of *β*_*x*_ is defined as the inverse of its variance, that is, ${\left(\text{Var}\left({\hat{\beta }}_{x}|\xi \right)\right)}^{-1}$. In the sequel, we will consider the efficiency of one design, *ξ*_1_, versus another design, *ξ*_2_, which is defined as $\text{Var}\left({\hat{\beta }}_{x}|\xi_{2}\right)/\text{Var}\left({\hat{\beta }}_{x}|\xi_{1}\right)$ and denoted as the relative efficiency. Since no closed-form expressions are available for the variances of the maximum likelihood (ML) estimator, asymptotic variances of the ML estimator were derived (Appendices A.1 and A.2).

The optimal allocation to treatments minimizes the variance of the estimator of *β*_treat_ in (1) or (2) and of *β*_treat×time_ in (3), given a fixed research budget. Note that changing the coding of the treatment factor or the time factor in (1)–(3), for instance into 1 versus −1 instead of 1 versus 0, will not affect the optimal allocation. Such a change of coding leads to a linear transformation of *β*_treat_ or *β*_treat×time_, and this will change the variance of their estimators only by a multiplicative constant. This implies that allocations that minimize the variance of the estimators do not depend on the coding of treatment and time.

To derive the optimal allocations under a budget restriction, we need to define a budget function. Let the costs involved with each subject in the parallel design be *c*_sp_ euros, in an extended parallel design be *c*_sep_ euros, and in a crossover design be *c*_sc_ euros. These costs may represent financial rewards given to subjects for participating in the trial but also the (average) costs of recruiting a subject. Furthermore, for treatments A and B there are, for each subject, costs *c*_A_ and *c*_B_, respectively, and each measurement may involve *c*_t_ euros. Finally, attached to each treatment sequence, there may be administration costs *c*_ts_.

In the case of allocation proportions *p*_A_ for treatment A and *p*_B_ = 1 − *p*_A_ for treatment B in a parallel design having *n*_p_ subjects, the following budget *C*^*∗*^ is required:

(4) $$\begin{array}{c} C^{*}=2c_{\text{ts}}+n_{\text{p}}\left(c_{\text{sp}}+c_{\text{t}}\right)+p_{\text{A}}n_{\text{p}}c_{\text{A}}+\left(1-p_{\text{A}}\right)n_{\text{p}}c_{\text{B}}. \end{array}$$

For the designs that we consider, this budget function can be reparametrized such that it is the same as the cost function given by Yuan and Zhou [8], thereby generalizing the cost function proposed by Brown [9] and Berger and Wong [4].

For an AB/BA crossover design, involving *n*_c_ subjects and allocation proportions *p*_AB_ for treatment sequence AB and *p*_BA_ = 1 − *p*_AB_ for treatment sequence BA, noting that each subject receives both treatment A and B and is measured twice, the following budget is required:

(5) $$\begin{array}{c} C^{*}=2c_{\text{ts}}+n_{\text{c}}c_{\text{sc}}+n_{\text{c}}c_{\text{A}}+n_{\text{c}}c_{\text{B}}+2n_{\text{c}}c_{\text{t}}. \end{array}$$

Finally, the required budget for an AA/BB design, involving *n*_ep_ subjects, with allocation proportions *p*_AA_ and *p*_BB_ = 1 − *p*_AA_ for the treatment sequences AA and BB, respectively, is as follows:

(6) $$\begin{array}{c} C^{*}=2c_{\text{ts}}+n_{\text{ep}}c_{\text{sep}}+2n_{\text{ep}}p_{\text{AA}}c_{\text{A}}+2n_{\text{ep}}\left(1-p_{\text{AA}}\right)c_{\text{B}}+{2n}_{\text{ep}}c_{\text{t}}. \end{array}$$

Note that, for the functions in (4)–(6), the budget may simply be the total number of observations involved in a study, by setting *c*_t_ = 1 and the other costs to 0. It can also represent the total number of subjects involved, by setting *c*_sp_ = *c*_sep_ = *c*_sc_ = 1 and the remaining costs to 0.

In what follows, we will assume that the subject-specific costs of the two-period designs are the same; that is, *c*_sep_ = *c*_sc_ = *c*_s_2p_. Since subjects in these designs receive two treatments and a washout period may be involved, these costs are very likely larger than those of a parallel design. We also assume that the subject-specific costs for the two-period designs will not exceed 2 times the subject-specific costs for the parallel design, so that *c*_sp_ ≤ *c*_s_2p_ ≤ 2*c*_sp_. Finally, since each design involves two treatment sequences, the administration costs are the same for each of the three designs considered, and thus, the budgets that are available for remaining costs are identical; that is, the budget *C* = *C*^*∗*^ − 2*c*_st_ is the same for each design.

### 3.1. Treatment Effect

For treatment effect estimation, the optimal allocations to the treatment sequences are derived in Appendix B. The optimal allocations and corresponding (asymptotic) variances of the treatment effect estimators are shown in the second and third column of Table 1, respectively. The optimal allocation ratios of the parallel and the extended parallel design depend on the costs and intraclass correlations: the more the expensive treatment A (or the cheaper treatment B) and the larger the intraclass correlation in treatment A (or the smaller the intraclass correlation in treatment B), the more the subjects have to be assigned to treatment B. The optimal allocation ratio for a crossover design is 1, which may be expected, since both groups receive both treatment A and B.

### 3.2. Treatment by Period Interaction Effect

In the case the treatment by period interaction effect is of primary interest, the optimal allocations can be derived along lines similar to the derivations for the treatment effect (Appendix B). The allocations and corresponding optimal variances are displayed in Table 1. Note that, similar to treatment effect estimation, the allocation ratio for a crossover design is 1, whereas the allocation ratio for an extended parallel design depends on the treatment costs and intraclass correlations, such that more persons are allocated to treatment sequence AA if the intraclass correlation of A decreases, the intraclass correlation of B increases, the costs of treatment A decrease, or the costs of treatment B increase.

## 4. Maximin Designs

Choosing the optimal allocation requires knowledge on the intraclass correlations *ρ*_A_ and *ρ*_B_ (remember that the variance ratio *ϕ* is fixed if *ρ*_A_ and *ρ*_B_ are given). Commonly, there is only limited knowledge on these parameters. A possible solution is the maximin strategy [4], consisting of 2 steps: (1) for each design determine the minimum efficiency of the effect estimator across the plausible ranges for the intraclass correlations *ρ*_A_ and *ρ*_B_ and (2) choose that design which maximizes this minimum efficiency. Such a design optimizes a worst case scenario and is called a *maximin design*. The maximin strategy implies choosing the design that minimizes the maximum variance of the estimator of the effect of interest. In determining sample sizes, choosing values for the intraclass correlations *ρ*_A_ and *ρ*_B_ within their plausible ranges (and thus a variance ratio *ϕ* within its plausible range) for which the variance is maximum will guarantee the desired power level also for all other values of these parameters. Moreover, the maximin design guarantees this power level at the lowest research costs. In what follows, we will refer to ranges of *ρ*_A_ and *ρ*_B_ that have lower bounds *ρ*_A_^L^ and *ρ*_B_^L^ and upper bounds *ρ*_A_^U^ and *ρ*_B_^U^, respectively.

### 4.1. Treatment Effect

From the asymptotic variances in Table 1, one can derive for which values of *ρ*_A_ and *ρ*_B_ (and thus for which value of the variance ratio *ϕ*), the variance of the treatment effect estimator is maximized. These derivations are given in Appendix C. The maximin parameter values and corresponding variances for the treatment effect estimator under optimal allocation to the treatments are shown in Table 2. The corresponding optimal allocations for the maximin designs are obtained by substituting the maximin parameter values of Table 2 into the allocation ratios as given in Table 1.

If for a parallel design the maximin value for the variance ratio *ϕ*^*∗*^ = (*c*_A_+*c*_t_+*c*_sp_)/(*c*_B_+*c*_t_+*c*_sp_) is within the plausible range for *ϕ*, that is, [*ρ*_B_^L^/*ρ*_A_^U^, *ρ*_B_^U^/*ρ*_A_^L^] and *c*_s_2p_ ≤ 2*c*_sp_, then a parallel design is always less efficient than a maximin crossover design. If for an extended parallel design the maximin value for one of the intraclass correlations is within the plausible range for the corresponding intraclass correlation, then also this design is less efficient than a maximin crossover design. For other scenarios, the relations between the maximin designs are more complicated, depending on the ranges for *ρ*_A_ and *ρ*_B_, the costs of treatments, subject recruitment, and measurement.

A systematic numerical evaluation was done to examine under what conditions the crossover design is the best choice in terms of efficiency. For *ρ*_A_ and *ρ*_B_, we consider ranges of width 0.10 (small), 0.30 (medium), and 0.60 (large). The lower bounds were {0.01, 0.05, 0.10, 0.15, 0.20,…}, where the largest possible lower bound was determined by the width of the range under consideration. For instance, if the range is 0.30 (medium), the largest lower bound for the intraclass correlation is 0.70. All combinations of small, medium, and large ranges for *ρ*_A_ and *ρ*_B_ were considered. The values of the variance ratio *ϕ* thus considered vary from 1/100 to 100. Since in most crossover trials, the intraclass correlation exceeds 0.30 [1–3, 10, 11], ranges with lower bounds of 0.30 or higher are empirically most relevant. The empirical evidence on the costs *c*_A_, *c*_B_, *c*_t_, *c*_sp_, and *c*_s_2p_ is scarce, and we thus choose costs covering a wide range of scenarios. Let CR_A_ = (*c*_A_ + *c*_t_)/c_sp_, CR_B_ = (*c*_B_ + *c*_t_)/c_sp_, and CR_p_ = *c*_s_2p_/*c*_sp_ (note that the relative efficiencies of the maximin designs depend only on these cost ratios). CR_A_ and CR_B_ take on the values 100, 20, 10, 1, 0.1, 0.05, and 0.01. For CR_p_, we consider 1 and 2.

If the costs of treatments are identical between the treatment arms, that is, CR_A_ = CR_B_, for most scenarios examined, the crossover maximin design turns out to be most efficient. For CR_p_ = 1 and CR_A_ = CR_B_ ≤ 1, the crossover design always is the most efficient. For CR_p_ = 1 and CR_A_ = CR_B_ > 1, or CR_p_ = 2, only if the lower bound of one of the intraclass correlations is 0.05 or lower and the ranges of the intraclass correlations do not overlap, the parallel design can become most efficient. Since in most empirical studies the intraclass correlations will exceed 0.05, this implies that, for equal costs of treatments, the crossover maximin design will almost always be the most efficient design.

In the case the treatment costs differ and CR_A_ ≤ 1 and CR_B_ ≤ 1, only in the case the lower bound of one of the intraclass correlations is 0.10 or smaller, the parallel or the extended parallel maximin design can become most efficient. The extended parallel design can only become most efficient if CR_p_ = 1. Hence, in all scenarios with unequal treatment costs and CR_A_ ≤ 1 and CR_B_ ≤ 1, for intraclass correlations of 0.15 or higher, the maximin crossover design is most efficient.

**Figure 1:**
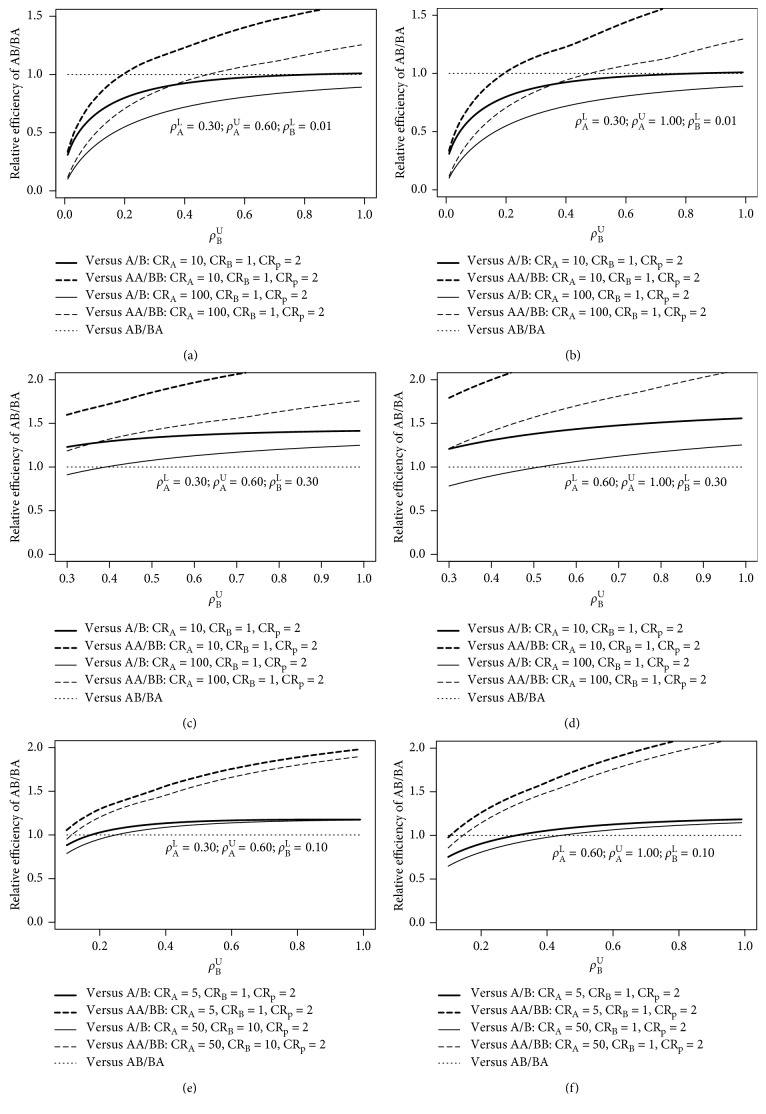
Relative efficiency for the treatment estimator of the AB/BA design versus the A/B and AA/BB designs as a function of the ranges for the intraclass correlations in treatment A (lower bound = *ρ*_A_^L^, upper bound = *ρ*_A_^U^) and B (lower bound = *ρ*_B_^L^, upper bound = *ρ*_B_^U^), in case of minimizing the research costs.

In the case the treatment costs differ and CR_A_ > 1 or CR_B_ > 1, the maximin crossover design is less often most efficient. For these cost scenarios, also for ranges of intraclass correlations exceeding 0.15, the maximin parallel and extended parallel design may become more efficient. This especially occurs if the costs of treatment A and lower bound of the range of *ρ*_A_ are both larger (or smaller) than the costs of treatment B and lower bound of *ρ*_B_, respectively. The efficiency improvement is large if treatment A is much more expensive than treatment B and if the costs of treatments and measurements are large compared to the subject-related costs. This is illustrated in Figure 1. The top row shows that if the costs of treatment A are larger than the costs of treatment B and the lower bound of *ρ*_A_ is larger than the lower bound of *ρ*_B_, a parallel design is most efficient, even up to an upper bound 1 of *ρ*_B_ if CR_A_ = 100. As can also be seen, the upper bound of *ρ*_A_ is not very relevant in terms of the relative efficiencies. The left plot of the middle row of Figure 1 shows that if the lower bounds of *ρ*_A_ and *ρ*_B_ are equal, then for almost all upper bounds of *ρ*_B_, the crossover design is most efficient. Again, as can be seen in the rightmost plot of the middle row, if the lower bound of *ρ*_A_ is higher than the lower bound of *ρ*_B_, then for higher upper bounds of *ρ*_B_, the parallel design is most efficient but to a lesser extent as compared to a smaller lower bound of *ρ*_B_. As is evident from the four subplots in the top and middle row, when increasing the ratio CR_A_/CR_B_, the crossover design becomes less efficient as compared to the other two designs. The subplots of the bottom row furthermore show that the crossover design also becomes less efficient compared to the other designs if CR_A_ and CR_B_ increase while the ratio CR_A_/CR_B_ remains constant. This illustrates that the efficiency of the other designs relative to the crossover design becomes larger if the costs of treatments and measurements are large compared to the subject-related costs. However, to summarize, if the treatment costs differ and CR_A_ > 1 or CR_B_ > 1, no simple rules of the thumb emerge and the most solid way to choose the most efficient design is just to calculate the maximin variances as given in Table 2.

Finally, if CR_p_ = 2, the maximin parallel design is consistently more efficient than the maximin extended parallel design (as is illustrated in Figure 1). If CR_p_ = 1, the maximin extended parallel design can also become more efficient than the maximin parallel design.

### 4.2. Treatment by Period Interaction Effect

The maximin parameter values and corresponding variances of the estimator of the treatment by period interaction effect are shown in Table 3. The derivations of these results can be done along lines similar to the derivations for the treatment effect estimator (Appendix C). The optimal allocation for the extended parallel design is obtained by substituting the maximin parameter values in the expression for the allocation ratio in Table 1. For a crossover design, the allocation ratio is 1. The maximin efficiency of an extended parallel design is always higher than that of a crossover design if the maximin value *ρ*_A_^*∗*^ is within the plausible range for *ρ*_*A*_. This follows from

(7) $$\begin{array}{c} \frac{4\left(c_{\text{A}}\left(1+\rho_{\text{B}}^{\text{L}}\right)+c_{\text{B}}\left(1-\rho_{\text{B}}^{\text{L}}\right)+2c_{\text{t}}+c_{\text{s\_}2\text{p}}\right)}{\left(1+\rho_{\text{B}}^{\text{L}}\right)}\left(\frac{\sigma_{y}^{2}}{C}\right)\leq 4\left(c_{\text{A}}+c_{\text{B}}+2c_{\text{t}}+c_{\text{s\_}2\text{p}}\right)\left(\frac{\sigma_{y}^{2}}{C}\right), \end{array}$$

where the right-hand side of the inequality in turn is smaller than the variance of a maximin crossover design (Table 3). The higher maximin efficiency of the extended parallel design can also be shown to hold if the maximin value *ρ*_B_^*∗*^ is within the plausible range for *ρ*_*B*_. Furthermore, if the variance maximizing values *ρ*_A_^*∗*^and *ρ*_B_^*∗*^ are outside the plausible ranges for *ρ*_A_ and *ρ*_B_, respectively, then values for *ρ*_A_ and *ρ*_B_ that coincide with one of the borders of their corresponding ranges should be chosen as values that maximize the variance. But in that case, even smaller variances result for the extended parallel design.

### 4.3. Maximin Designs That Minimize the Number of Subjects and Number of Measurements

As noted in Section 3, by setting *c*_sp_ = *c*_sep_ = *c*_sc_ = 1 and the remaining costs to 0 in (4)–(6), the budget is simply the total number of subjects involved in a study, and by setting *c*_t_ = 1 and the other costs to 0, the budget reduces to the total number of measurements involved. When the budget is the total sample size and interest is in estimating the treatment effect, it can be proven, based on the formulas in Table 2, that a maximin crossover design requires less subjects than a maximin parallel design. From an extensive numerical evaluation analogous to the one of Section 4.1, a maximin crossover design also appears to require less subjects than a maximin extended parallel design.

When minimizing the number of measurements, the numerical evaluation shows again that the maximin crossover design is the best choice provided the lower bounds of both intraclass correlations are 0.10 or higher. In other cases also a maximin parallel design may minimize the total number of measurements. Since in most crossover trials the intraclass correlation exceeds 0.30 [1–3, 10, 11], in practice, this implies that the maximin crossover trial also is the best choice when minimizing the number of measurements.

In the case, interest is in the treatment by period interaction, Section 4.2 showed a maximin extended parallel design to be more efficient and thus also to require less budget than a maximin crossover trial. In the special case where the number of subjects or the total number of measurements are minimized, the maximin extended parallel design will therefore also outperform the maximin crossover design.

## 5. Monte Carlo Evaluation of the Power of Maximin Designs

The efficiencies as derived for the maximin designs are based on the asymptotic variance of the ML estimator, $\text{Var}\left({\hat{\beta }}_{x}|\xi \right)$. For sufficiently large numbers of subjects, the relation between the asymptotic variance of the ML estimator and the power level 1 − *γ* to detect a treatment effect in a two-tailed test with type I error rate *α* can be approximated as follows:

(8) $$\begin{array}{c} \text{Var}\left({\hat{\beta }}_{x}\;|\;\xi \right)={\left(\frac{\beta_{x}}{z_{1-\alpha /2}+z_{1-\gamma }}\right)}^{2}, \end{array}$$

where *z*_1 − *α*/2_ and *z*_1−*γ*_ are the 100 (1 − *α*/2) and 100 (1 − *γ*) percentiles of the standard normal distribution. For small sample sizes calculated by (8), corrections are needed [12, 13]. For each of the three designs, these corrections will be applied. We will examine to what extent the differences between designs in asymptotic efficiencies translate into corresponding differences in power levels for small samples. Also, when planning sample sizes based on the asymptotic variances, we can check whether the commonly used power levels of 80% or 90% are realized in the case of small samples.

For the treatment effect estimator, the following expression for the required number of subjects results for a crossover design with optimal allocation:

(9) $$\begin{array}{c} n_{\text{c}}={\left(z_{1-\alpha /2}+z_{1-\gamma }\right)}^{2}\left(1-\frac{2\rho_{\text{A}}\rho_{\text{B}}}{\rho_{\text{A}}+\rho_{\text{B}}}\right)\left(\frac{\sigma_{\text{A}}^{2}+\sigma_{\text{B}}^{2}}{\beta_{\text{treat}}^{2}}\right). \end{array}$$

If we let ES = $\beta_{\text{treat}}/\sqrt{0.5\left(\sigma_{\text{A}}^{2}+\sigma_{\text{B}}^{2}\right)}$ be the effect size based on the outcome variances in the treatment and control arm (cf. [14]), then (9) can be rewritten as follows:

(10) $$\begin{array}{c} n_{\text{c}}=2{\left(\frac{z_{1-\alpha /2}+z_{1-\gamma }}{\text{ES}}\right)}^{2}\left(1-\frac{2\rho_{\text{A}}\rho_{\text{B}}}{\rho_{\text{A}}+\rho_{\text{B}}}\right). \end{array}$$

Note that, in the case of a maximin design, the expression is the same as (10), however, with *ρ*_A_^L^ and *ρ*_B_^L^ being substituted for *ρ*_A_ and *ρ*_B_, respectively. Similar rewritings of the sample sizes in terms of the effect size are possible for the parallel and extended parallel design, respectively:

(11) $$\begin{array}{c} n_{\text{p}}=2{\left(\frac{z_{1-\alpha /2}+z_{1-\gamma }}{\text{ES}}\right)}^{2}\left(1+\left(\frac{c_{\text{A}}+c_{\text{B}}+2c_{\text{t}}+2c_{\text{sp}}}{\sqrt{\left(c_{\text{A}}+c_{\text{t}}+c_{\text{sp}}\right)\left(c_{\text{B}}+c_{\text{t}}+c_{\text{sp}}\right)}}\right)\left(\sqrt{\frac{\rho_{\text{B}}}{\rho_{\text{A}}+\rho_{\text{B}}}}\sqrt{\frac{\rho_{\text{A}}}{\rho_{\text{A}}+\rho_{\text{B}}}}\right)\right),\\ n_{\text{ep}}={\left(\frac{z_{1-\alpha /2}+z_{1-\gamma }}{\text{ES}}\right)}^{2}\left(1+\frac{2\rho_{\text{A}}\rho_{\text{B}}}{\rho_{\text{A}}+\rho_{\text{B}}}+\left(\frac{2c_{\text{A}}+2c_{\text{B}}+4c_{\text{t}}+2c_{\text{s\_}2\text{p}}}{\sqrt{\left(2c_{\text{A}}+2c_{\text{t}}+c_{\text{s\_}2\text{p}}\right)\left(2c_{\text{B}}+2c_{\text{t}}+c_{\text{s\_}2\text{p}}\right)}}\right)\left(\sqrt{\frac{\rho_{\text{B}}\left(1+\rho_{\text{A}}\right)}{\rho_{\text{A}}+\rho_{\text{B}}}}\sqrt{\frac{\rho_{\text{A}}\left(1+\rho_{\text{B}}\right)}{\rho_{\text{A}}+\rho_{\text{B}}}}\right)\right). \end{array}$$

The choices to be made for *ρ*_A_ and *ρ*_B_ in the case of maximin versions of the parallel and extended parallel design are determined by the conditions as formulated in Table 2.

In the case of the treatment by period interaction effect, the following expression for the required number of subjects can be derived for a crossover design with optimal allocation:

(12) $$\begin{array}{c} n_{\text{c}}=8{\left(\frac{z_{1-\alpha /2}+z_{1-\gamma }}{\text{ES}}\right)}^{2}\left(1+\frac{2\rho_{\text{A}}\rho_{\text{B}}}{\rho_{\text{A}}+\rho_{\text{B}}}\right), \end{array}$$

where ES = $\beta_{\text{treat}\times \text{time}}/\sqrt{0.5\left(\sigma_{\text{A}}^{2}+\sigma_{\text{B}}^{2}\right)}$. In the case of a maximin design, the expression is the same as (12), however, with *ρ*_A_^U^ and *ρ*_B_^U^ being substituted for *ρ*_A_ and *ρ*_B_, respectively. The expression for the sample size of an extended parallel design, when allocating optimally, can be written as follows:

(13) $$\begin{array}{c} n_{\text{ep}}=4{\left(\frac{z_{1-\alpha /2}+z_{1-\gamma }}{\text{ES}}\right)}^{2}\left(1-\frac{2\rho_{\text{A}}\rho_{\text{B}}}{\rho_{\text{A}}+\rho_{\text{B}}}+\left(\frac{2c_{\text{A}}+2c_{\text{B}}+4c_{\text{t}}+2c_{\text{s\_}2\text{p}}}{\sqrt{\left(2c_{\text{A}}+2c_{\text{t}}+c_{\text{s\_}2\text{p}}\right)\left(2c_{\text{B}}+2c_{\text{t}}+c_{\text{s\_}2\text{p}}\right)}}\right)\left(\sqrt{\frac{\rho_{\text{B}}\left(1-\rho_{\text{A}}\right)}{\rho_{\text{A}}+\rho_{\text{B}}}}\sqrt{\frac{\rho_{\text{A}}\left(1-\rho_{\text{B}}\right)}{\rho_{\text{A}}+\rho_{\text{B}}}}\right)\right). \end{array}$$

The choices to be made for *ρ*_A_ and *ρ*_B_ in the case of a maximin extended parallel design are determined by the conditions formulated in Table 3.

Since maximin designs only require information on plausible ranges of model parameters, they are more practical than optimal designs. In what follows, we will therefore examine through a Monte Carlo simulation the power for maximin designs in the case of small sample sizes. First, we will discuss the factors that are varied and motivate the choices made for these factors in determining the simulation scenarios.

### 5.1. Choice of Ranges/Values for Relevant Factors

#### 5.1.1. Effect Sizes

The effect size, ES, is commonly categorized into small (0.2), medium (0.5), and large (0.8) [14]. Being primarily interested in the small sample performance of (9)–(13), we only will consider ES = 0.8, leading to the smallest sample sizes.

#### 5.1.2. Costs

The empirical evidence on costs is rather scarce, but we will choose the costs such that they imply minimizing the sample size of a study (i.e., *c*_A_ = *c*_B_ = *c*_t_ = 0 and *c*_sp_ = *c*_s_2p_ = 1).

#### 5.1.3. Intraclass Correlations

The ranges for *ρ*_A_ and *ρ*_B_ are identical to the ranges of the numerical evaluation of Section 4.1. Since we are interested in the small sample performance, for each design, we consider that pair of ranges across all combinations of ranges for the intraclass correlations (i.e., small-small, medium-medium, large-large, small-medium, small-large, and medium-large) that lead to the smallest sample sizes. Since this each time turns out to be a pair from the small-small category, the same was done for all pairs of medium and large ranges, which will be used more often in practice. For each design, the two resulting pairs of ranges of intraclass correlation are displayed in the two leftmost columns of Table 4 and the Table in Appendix D.

#### 5.1.4. Power Level and Type I Error Rate

In sample size planning commonly used power levels are 80% and 90% in a two-tailed test with either a 5% or a 1% type I error rate. Focusing on the small sample performance, we will consider 80% power in a two-tailed test with a 5% type I error rate. For small sample sizes derived from the standard normal distribution (as in (9)–(13)), corrections are needed that turn out to depend on the type I error rate [12, 13]. For this reason, we will also study a 1% type I error rate.

### 5.2. Simulation Procedure and Testing Methods

For each of the 20 simulation scenarios, 25,000 data sets were generated. To distinguish chance deviations of the simulated power from systematic deviations, a 95% predictive interval was calculated. For a nominal power *π*, this is defined as [$\pi +z_{0.025}\sqrt{\pi \left(1-\pi \right)/N\text{sim}}$, $\pi +z_{0.975}\sqrt{\pi \left(1-\pi \right)/N\text{sim}}$], where *z*_0.025_ and *z*_0.975_ are the 2.5 and 97.5 percentiles of the standard normal distribution and *N*sim is the number of simulations. Since the nominal power *π* = 0.80 and *N*sim = 25,000, the 95% predictive interval is [0.795, 0.805].

For a test of the treatment effect, the data generated for the crossover design were analyzed with a two-sample *t*-test on the difference scores obtained by subtracting the two measurements for each subject. The model in (2) implies homogeneity of variances for these difference scores, so that a pooled variance *t*-test was applied. The data generated for the parallel design were simply analyzed by a two-sample *t*-test on the original scores, whereas the data for the extended parallel design were analyzed with a two-sample *t*-test on the scores averaged across both measurements. For these parallel designs, (1) and (2) imply that the analyzed scores may have variances differing between groups, so that an unpooled variance *t*-test was applied. For the treatment by period interaction effect, the data generated for the crossover design were analyzed with a two-sample (pooled variance) *t*-test on the scores averaged for each subject across both measurements, whereas the data generated for the extended parallel design were analyzed with a two-sample (unpooled variance) *t*-test on the differences between the two measurements (3). These different *t*-tests follow for each of the designs (involving equal numbers of measurements per subject) from the analysis models in (1)–(3) and do not require asymptotic assumptions.

In calculating the required sample size, (9)–(11) were used, when interest is in testing the treatment effect, and (12) and (13) were used, when interest is in testing the treatment by period interaction. The optimal allocations for each design are given in Table 1, taking the maximin values for *ρ*_A_ and *ρ*_B_ as determined from Tables 2 and 3 for the treatment main and treatment by period interaction effect, respectively. Since the outcome variances are unknown, the calculated sample sizes were subsequently corrected. For an unpooled variance *t*-test, if the number of persons per arm is 8 or more, sufficient corrections for two-tailed tests are 2 extra persons per arm if *α* = 0.05 and 4 extra persons per arm if *α* = 0.01. With less than 8 persons in one or both arms, sufficient corrections are 3 extra persons per arm if *α* = 0.05 and 4 extra persons per arm if *α* = 0.01 ([13], p. 568). For a pooled variance *t*-test, we only need to add 1 person per arm if *α* = 0.05 (two-tailed) and 2 persons per arm if *α* = 0.01 (two-tailed) ([12], p. 1216-1217). These are sufficient corrections for planned powers of 80% and 90%. The simulations, statistical tests, and power calculations were done in *R*, version 3.1.3 [15].

### 5.3. Results

As can be seen in Table 4 and Appendix D, for all sample size-design combinations that should yield 80% power, the simulated powers were either within or above the 95% predictive intervals. This indicates that the asymptotic results, supplemented with simple correction rules for using the standard normal distribution, yield sample sizes that guarantee the desired level of power. The realized power levels generally are higher than 80%, since the small sample corrections are sufficient and in some cases smaller corrections would have been appropriate [12, 13].

The power differences between the designs can become rather large and are in line with the asymptotic relative efficiencies. For the examples of Table 4, the crossover design always is most efficient and in the simulation also has the highest power. Additional simulations show that similar conclusions can be drawn for ranges of intraclass correlations for which the crossover design is not most efficient. As expected, when testing the treatment by period interaction, the extended parallel design has more power than the crossover design (Appendix D).

## 6. Application in Planning a Trial

Suppose one would like to perform a randomized trial on the effectiveness of indacaterol versus tiotropium, among subjects suffering from chronic obstructive pulmonary disease, similar to Donohue et al. [16]. After 12 weeks of treatment, one plans to evaluate the effect of 18 *μ*g of tiotropium versus 300 *μ*g of indacaterol on the bronchodilator efficacy of 24 h postdose forced expiratory volume in 1 s (FEV1 in mL). The variance of FEV1 in the indacaterol and the tiotropium conditions in the study by Donohue et al. [16] differed significantly from each other, their ratio being 1.58. No information was available on the intraclass correlations and the research costs. Suppose reasonable guesses on the intraclass correlations are *ρ*_A_ ∈ [0.10, 0.70] for indacaterol and *ρ*_B_ ∈ [0.30, 0.90] for tiotropium. If one aims at minimizing the total sample size (so we set *c*_A_ = *c*_B_ = *c*_t_ = 0 and *c*_sp_ = *c*_s_2p_ = 1), the maximin crossover design is more efficient than the AA/BB and A/B designs requiring only 48% and 43%, respectively, of the number of subjects of these designs. To be able to detect a medium effect (ES = 0.5, see below (9)) with 80% power in a two-tailed test with a 5% type I error rate, taking as maximin parameter values *ρ*_A_^*∗*^ = 0.10 and *ρ*_B_^*∗*^ = 0.30 in (10), 54 subjects are needed. Since the sample size calculation in (10) is based on the standard normal, whereas the test statistic follows a *t*-distribution, we add 1 subject to each treatment sequence [12], yielding a total sample size of 56 subjects with 28 subjects being allocated to each of the two treatment sequences of the crossover design.

## 7. Conclusion and Discussion

We examined the asymptotic efficiency of the ML estimator of the treatment and the treatment by period interaction effect for three two-treatment designs: a parallel, an extended parallel, and a crossover design. For a flexible cost function, the optimal allocations to the treatment sequences and corresponding optimal efficiencies were derived. Since commonly the intraclass correlations for each of the treatments and the ratio of treatment-dependent variances are not precisely known, also maximin designs were derived, which guarantee a power level across plausible ranges of values for the intraclass correlations at the lowest costs.

When interested in testing the main effects of the treatments, the relations between the efficiencies of the maximin versions of the A/B, AB/BA, and AA/BB designs depend on assumed ranges of the intraclass correlations, on the costs of the treatments and the costs of recruiting and measuring subjects. A numerical investigation shows that if A and B are equally expensive or the sum of the costs of one treatment and measurement per person are less than the remaining subject-specific costs (such as recruitment costs), then the crossover design is most efficient for ranges of intraclass correlations starting at 0.15 or higher. In other cost scenarios, also for ranges of the intraclass correlations above 0.15, the parallel design or its extended version may become most efficient. Then, the efficiency relations are complicated, and the most efficient design is best determined by the results in Table 2. For the treatment by period interaction, however, the maximin AA/BB trial is proven to be more efficient than the maximin AB/BA design.

Since the efficiency comparisons of the maximin designs were based on asymptotic variances, a Monte Carlo simulation study was done for small samples. After applying correction factors in sample size planning based on the standard normal distribution, it was shown that (a) the asymptotic relative efficiencies translate into corresponding relative power levels and (b) power levels targeted in sample size planning are realized. This illustrates the practical utility of these results for sample size calculation.

If prerandomization measurements of the outcome variable are available, these could be included as covariates in the analysis [1]. Adding covariates in a randomized trial will not change the treatment effect of interest but will lead to a reduction of the intercept variance and thus of the intraclass correlations [3]. Provided that the costs of prerandomization measurements are the same for all designs (and there are no missing values on these prerandomization covariates), the results of the present study also apply.

The present study did not consider carry over in deriving optimal and maximin designs. If there is *self carry over*, that is, carry over from a treatment onto itself, this implies that steady-state did not yet occur in the first period, and then the total treatment effect would be the relevant effect, that is, the direct effect in the first period plus the carry over effect in the second period [5]. If there is self carry over, one-period designs and the AB/BA design are not suitable, as they do not allow for estimating the total treatment effect, leaving only the AA/BB design as a suitable option. There may also be *steady-state carry over*, which can only occur if there is a switch of treatments [17]. Such carry over would affect the efficiency of the crossover design. Although one commonly tries to avoid carry over, examining to what extent steady-state carry over affects the relative efficiency of the maximin crossover design would be an interesting issue for further research.

## Figures and Tables

**Table 1:** Optimal allocation ratios and corresponding variances of the ML estimator of the treatment effect and the treatment by period interaction for different designs under heterogeneity of outcome variances and costs.

Treatment effect		
Design	Allocation ratio (*n*_1_/*n*_2_)	$\text{Var}\left({\hat{\beta }}_{\text{treat}}\right)$
Crossover design	1	(*c*_A_+*c*_B_+2*c*_t_+*c*_s_2p_)(1 − ((2*ρ*_A_*ρ*_B_)/(*ρ*_A_+*ρ*_B_)))*σ*_*y*_^2^/*C*
Parallel design	$\sqrt{\left(\rho_{\text{B}}/\rho_{\text{A}}\right)\left(\left(c_{\text{B}}+c_{\text{t}}+c_{\text{sp}}\right)/\left(c_{\text{A}}+c_{\text{t}}+c_{\text{sp}}\right)\right)}$	${\left(\sqrt{\left(c_{\text{A}}+c_{\text{t}}+c_{\text{sp}}\right)\left(\rho_{\text{B}}/\left(\rho_{\text{A}}+\rho_{\text{B}}\right)\right)}+\sqrt{\left(c_{\text{B}}+c_{\text{t}}+c_{\text{sp}}\right)\left(\rho_{\text{A}}/\left(\rho_{\text{A}}+\rho_{\text{B}}\right)\right)}\right)}^{2}\left(\sigma_{y}^{2}/C\right)$
Extended parallel design	$\sqrt{\left(\rho_{\text{B}}\left(1+\rho_{\text{A}}\right)/\left(\rho_{\text{A}}\left(1+\rho_{\text{B}}\right)\right)\right)\times \left(\left(2\left(c_{\text{B}}+c_{\text{t}}\right)+c_{\text{s\_}2\text{p}}\right)/\left(2\left(c_{\text{A}}+c_{\text{t}}\right)+c_{\text{s\_}2\text{p}}\right)\right)}$	${\left(\sqrt{\left(2\left(c_{\text{A}}+c_{\text{t}}\right)+c_{\text{s\_}2\text{p}}\right)\left(\left(\rho_{\text{B}}\left(1+\rho_{\text{A}}\right)\right)/\left(\rho_{\text{A}}+\rho_{\text{B}}\right)\right)}+\sqrt{\left(2\left(c_{\text{B}}+c_{\text{t}}\right)+c_{\text{s\_}2\text{p}}\right)\left(\left(\rho_{\text{A}}\left(1+\rho_{\text{B}}\right)\right)/\left(\rho_{\text{A}}+\rho_{\text{B}}\right)\right)}\right)}^{2}\sigma_{y}^{2}/\left(2C\right)$

Treatment by period interaction		
Design	Allocation ratio (*n*_1_/*n*_2_)	$\text{Var}\left({\hat{\beta }}_{\text{treat}\times \text{time}}\right)$

Crossover design	1	4(*c*_A_+*c*_B_+2*c*_t_+*c*_s_2p_)(1+2*ρ*_A_*ρ*_B_/(*ρ*_A_+*ρ*_B_))(*σ*_*y*_^2^/*C*)
Extended parallel design	$\sqrt{\left(\rho_{\text{B}}\left(1-\rho_{\text{A}}\right)/\left(\rho_{\text{A}}\left(1-\rho_{\text{B}}\right)\right)\right)\times \left(2\left(c_{\text{B}}+c_{\text{t}}\right)+c_{\text{s\_}2\text{p}}\right)/\left(2\left(c_{\text{A}}+c_{\text{t}}\right)+c_{\text{s\_}2\text{p}}\right)}$	$2{\left(\sqrt{\left(2\left(c_{\text{A}}+c_{\text{t}}\right)+c_{\text{s\_}2\text{p}}\right)\left(\left(\rho_{\text{B}}\left(1-\rho_{\text{A}}\right)\right)/\left(\rho_{\text{A}}+\rho_{\text{B}}\right)\right)}+\sqrt{\left(2\left(c_{\text{B}}+c_{\text{t}}\right)+c_{\text{s\_}2\text{p}}\right)\left(\left(\rho_{\text{A}}\left(1-\rho_{\text{B}}\right)\right)/\left(\rho_{\text{A}}+\rho_{\text{B}}\right)\right)}\right)}^{2}\left(\sigma_{y}^{2}/C\right)$

*Note*. *n*_1_: sample size for A (parallel design), AB (crossover design), or AA (extended parallel design) sequence; *n*_2_: sample size for B (parallel design), BA (crossover design), or BB (extended parallel design) sequence; *σ*_*y*_^2^=*σ*_A_^2^+*σ*_B_^2^.

**Table 2:** Values for the parameters and variances of the treatment effect estimator for each of the maximin designs.

Design	Maximin parameter values	$\text{Var}\left({\hat{\beta }}_{\text{treat}}\right)$ for the maximin design
Crossover design	*ρ*_A_^L^, *ρ*_B_^L^	(*c*_A_+*c*_B_+2*c*_t_+*c*_s_2p_)(1 − 2*ρ*_A_^L^*ρ*_B_^L^/(*ρ*_A_^L^+*ρ*_B_^L^))(*σ*_*y*_^2^/*C*)

Parallel design	If (*ρ*_B_^L^/*ρ*_A_^U^) ≤ (*c*_A_+*c*_t_+*c*_sp_)/(*c*_B_+*c*_t_+*c*_sp_) ≤ (*ρ*_B_^U^/*ρ*_A_^L^), choose (*ρ*_B_/*ρ*_A_)=(*c*_A_+*c*_t_+*c*_sp_)/(*c*_B_+*c*_t_+*c*_sp_)	(*c*_A_+*c*_B_+2(*c*_t_+*c*_sp_))(*σ*_*y*_^2^/*C*)
	If (*c*_A_+*c*_t_+*c*_sp_)/(*c*_B_+*c*_t_+*c*_sp_) > (*ρ*_B_^U^/*ρ*_A_^L^), then *ρ*_A_^L^ and *ρ*_B_^U^	${\left(\sqrt{\left(c_{\text{A}}+c_{\text{t}}+c_{\text{sp}}\right)\left(\rho_{\text{B}}^{\text{U}}/\left(\rho_{\text{A}}^{\text{L}}+\rho_{\text{B}}^{\text{U}}\right)\right)}+\sqrt{\left(c_{\text{B}}+c_{\text{t}}+c_{\text{sp}}\right)\left(\rho_{\text{A}}^{\text{L}}/\left(\rho_{\text{A}}^{\text{L}}+\rho_{\text{B}}^{\text{U}}\right)\right)}\right)}^{2}\left(\sigma_{y}^{2}/C\right)$
	If (*c*_A_+*c*_t_+*c*_sp_)/(*c*_B_+*c*_t_+*c*_sp_) < (*ρ*_B_^L^/*ρ*_A_^U^), then *ρ*_A_^U^ and *ρ*_B_^L^	${\left(\sqrt{\left(c_{\text{A}}+c_{\text{t}}+c_{\text{sp}}\right)\left(\rho_{\text{B}}^{\text{L}}/\left(\rho_{\text{A}}^{\text{U}}+\rho_{\text{B}}^{\text{L}}\right)\right)}+\sqrt{\left(c_{\text{B}}+c_{\text{t}}+c_{\text{sp}}\right)\left(\rho_{\text{A}}^{\text{U}}/\left(\rho_{\text{A}}^{\text{U}}+\rho_{\text{B}}^{\text{L}}\right)\right)}\right)}^{2}\left(\sigma_{y}^{2}/C\right)$

Extended parallel design	If (1/*ρ*_A_^U^)+1 ≤ ((*λ*(1 − *ρ*_B_^U^)^2^)/((1+*ρ*_B_^U^)*ρ*_B_^U^)) ≤ (1/*ρ*_A_^L^)+1, then *ρ*_A_^*∗*^=(*ρ*_B_^U^(1+*ρ*_B_^U^))/(*λ*(1 − *ρ*_B_^U^)^2^ − *ρ*_B_^U^(1+*ρ*_B_^U^)), *ρ*_B_^U^	(*c*_A_+*c*_B_((1+*ρ*_B_^U^)/(1 − *ρ*_B_^U^))+((2*c*_t_+*c*_s_2p_)/(1 − *ρ*_B_^U^)))(*σ*_*y*_^2^/*C*)
	else if (1/*ρ*_B_^U^)+1 ≤ (((1 − *ρ*_A_^U^)^2^)/(*λ*(1+*ρ*_A_^U^)*ρ*_A_^U^)) ≤ (1/*ρ*_B_^L^)+1, then *ρ*_A_^U^, *ρ*_B_^*∗*^=(*λρ*_A_^U^(1+*ρ*_A_^U^))/((1 − *ρ*_A_^U^)^2^ − *λρ*_A_^U^(1+*ρ*_A_^U^)),	(*c*_A_((1+*ρ*_A_^U^)/(1 − *ρ*_A_^U^))+*c*_B_+((2*c*_t_+*c*_s_2p_)/(1 − *ρ*_A_^U^)))(*σ*_*y*_^2^/*C*)
	else if (*λ*(1 − *ρ*_B_^U^)^2^)/((1+*ρ*_B_^U^)*ρ*_B_^U^) < (1/*ρ*_A_^U^)+1, and (1 − *ρ*_A_^U^)^2^/(*λ*(1+*ρ*_A_^U^)*ρ*_A_^U^) < (1/*ρ*_B_^U^)+1, then *ρ*_A_^U^, *ρ*_B_^U^	${\left(\sqrt{\left(2\left(c_{\text{A}}+c_{\text{t}}\right)+c_{\text{s\_}2\text{p}}\right)\left(\rho_{\text{B}}^{\text{U}}\left(1+\rho_{\text{A}}^{\text{U}}\right)/\left(\rho_{\text{A}}^{\text{U}}+\rho_{\text{B}}^{\text{U}}\right)\right)}+\sqrt{\left(2\left(c_{\text{B}}+c_{\text{t}}\right)+c_{\text{s\_}2\text{p}}\right)\left(\rho_{\text{A}}^{\text{U}}\left(1+\rho_{\text{B}}^{\text{U}}\right)/\left(\rho_{\text{A}}^{\text{U}}+\rho_{\text{B}}^{\text{U}}\right)\right)}\right)}^{2}\left(\sigma_{y}^{2}/\left(2C\right)\right)$
	else if ((*λ*(1 − *ρ*_B_^U^)^2^)/((1+*ρ*_B_^U^)*ρ*_B_^U^)) < (1/*ρ*_A_^U^)+1, then *ρ*_A_^U^, *ρ*_B_^L^	${\left(\sqrt{\left(2\left(c_{\text{A}}+c_{\text{t}}\right)+c_{\text{s\_}2\text{p}}\right)\left(\rho_{\text{B}}^{\text{L}}\left(1+\rho_{\text{A}}^{\text{U}}\right)/\left(\rho_{\text{A}}^{\text{U}}+\rho_{\text{B}}^{\text{L}}\right)\right)}+\sqrt{\left(2\left(c_{\text{B}}+c_{\text{t}}\right)+c_{\text{s\_}2\text{p}}\right)\left(\rho_{\text{A}}^{\text{U}}\left(1+\rho_{\text{B}}^{\text{L}}\right)/\left(\rho_{\text{A}}^{\text{U}}+\rho_{\text{B}}^{\text{L}}\right)\right)}\right)}^{2}\left(\sigma_{y}^{2}/\left(2C\right)\right)$
	else *ρ*_A_^L^, *ρ*_B_^U^	${\left(\sqrt{\left(2\left(c_{\text{A}}+c_{\text{t}}\right)+c_{\text{s\_}2\text{p}}\right)\left(\rho_{\text{B}}^{\text{U}}\left(1+\rho_{\text{A}}^{\text{L}}\right)/\left(\rho_{\text{A}}^{\text{L}}+\rho_{\text{B}}^{\text{U}}\right)\right)}+\sqrt{\left(2\left(c_{\text{B}}+c_{\text{t}}\right)+c_{\text{s\_}2\text{p}}\right)\left(\rho_{\text{A}}^{\text{L}}\left(1+\rho_{\text{B}}^{\text{U}}\right)/\left(\rho_{\text{A}}^{\text{L}}+\rho_{\text{B}}^{\text{U}}\right)\right)}\right)}^{2}\left(\sigma_{y}^{2}/\left(2C\right)\right)$

*Note.σ*_*y*_^2^=*σ*_A_^2^+*σ*_B_^2^; *λ*=(2*c*_A_+2*c*_t_+*c*_s_2p_)/(2*c*_B_+2*c*_t_+*c*_s_2p_).

**Table 3:** Values for the parameters and variances of the treatment by period interaction effect estimator for each of the maximin designs.

Design	Maximin parameter values	$\text{Var}\left({\hat{\beta }}_{\text{treat}\times \text{time}}\right)$
Crossover design	*ρ*_A_^U^, *ρ*_B_^U^	(4(*c*_A_+*c*_B_+2*c*_t_+*c*_s_2p_)(1+((2*ρ*_A_^U^*ρ*_B_^U^)/(*ρ*_A_^U^+*ρ*_B_^U^))))(*σ*_*y*_^2^/*C*)

Extended parallel design	If(1/*ρ*_A_^U^) − 1 ≤ ((*λ*(1+*ρ*_B_^L^)^2^)/((1 − *ρ*_B_^L^)*ρ*_B_^L^)) ≤ (1/*ρ*_A_^L^) − 1, then *ρ*_A_^*∗*^=(*ρ*_B_^L^(1 − *ρ*_B_^L^))/(*λ*(1+*ρ*_B_^L^)^2^+*ρ*_B_^L^(1 − *ρ*_B_^L^)), *ρ*_B_^L^,	((4(*c*_A_(1+*ρ*_B_^L^)+*c*_*B*_(1 − *ρ*_B_^L^)+2*c*_t_+*c*_s_2p_))/(1+*ρ*_B_^L^))(*σ*_*y*_^2^/*C*)
	$\begin{array}{c} \text{else if }\left(1/\rho_{\text{B}}^{\text{U}}\right)-1\leq \left(\left({\left(1+\rho_{\text{A}}^{\text{L}}\right)}^{2}\right)/\left(\lambda \left(1-\rho_{\text{A}}^{\text{L}}\right)\rho_{\text{A}}^{\text{L}}\right)\right)\leq \left(1/\rho_{\text{B}}^{\text{L}}\right)-1,\text{then }\rho_{\text{A}}^{\text{L}},\rho_{\text{B}}^{*}=\left(\lambda \rho_{\text{A}}^{\text{L}}\left(1-\rho_{\text{A}}^{\text{L}}\right)\right)/\left({\left(1+\rho_{\text{A}}^{\text{L}}\right)}^{2}+\lambda \rho_{\text{A}}^{\text{L}}\left(1-\rho_{\text{A}}^{\text{L}}\right)\right), \end{array}$	((4(*c*_A_(1 − *ρ*_A_^L^)+*c*_*B*_(1+*ρ*_A_^L^)+2*c*_t_+*c*_s_2p_))/(1+*ρ*_A_^L^))(*σ*_*y*_^2^/*C*)
	$\begin{array}{c} \text{else if }\left(\lambda {\left(1+\rho_{\text{B}}^{\text{L}}\right)}^{2}\right)/\left(\left(1-\rho_{\text{B}}^{\text{L}}\right)\rho_{\text{B}}^{\text{L}}\right)>\left(1/\rho_{\text{A}}^{\text{L}}\right)-1\text{and }{\left(1+\rho_{\text{A}}^{\text{L}}\right)}^{2}/\left(\lambda \left(1-\rho_{\text{A}}^{\text{L}}\right)\rho_{\text{A}}^{\text{L}}\right)>\left(1/\rho_{\text{B}}^{\text{L}}\right)-1,\text{then }\rho_{\text{A}}^{\text{L}},\rho_{\text{B}}^{\text{L}} \end{array}$	$2{\left(\sqrt{\left(\left(2\left(c_{\text{A}}+c_{\text{t}}\right)+c_{\text{s\_}2\text{p}}\right)\left(\left(\rho_{\text{B}}^{\text{L}}\left(1-\rho_{\text{A}}^{\text{L}}\right)\right)/\left(\rho_{\text{A}}^{\text{L}}+\rho_{\text{B}}^{\text{L}}\right)\right)\right)}+\sqrt{\left(\left(2\left(c_{\text{B}}+c_{\text{t}}\right)+c_{\text{s\_}2\text{p}}\right)\left(\rho_{\text{A}}^{\text{L}}\left(1-\rho_{\text{B}}^{\text{L}}\right)/\left(\rho_{\text{A}}^{\text{L}}+\rho_{\text{B}}^{\text{L}}\right)\right)\right)}\right)}^{2}\left(\sigma_{y}^{2}/C\right)$
	$\begin{array}{c} \text{else if }\left(\left(\lambda {\left(1+\rho_{\text{B}}^{\text{L}}\right)}^{2}\right)/\left(\left(1-\rho_{\text{B}}^{\text{L}}\right)\rho_{\text{B}}^{\text{L}}\right)\right)<\left(1/\rho_{\text{A}}^{\text{U}}\right)-1,\text{then }\rho_{\text{A}}^{\text{U}},\rho_{\text{B}}^{\text{L}} \end{array}$	$2{\left(\sqrt{\left(\left(2\left(c_{\text{A}}+c_{\text{t}}\right)+c_{\text{s\_}2\text{p}}\right)\left(\left(\rho_{\text{B}}^{\text{L}}\left(1-\rho_{\text{A}}^{\text{U}}\right)\right)/\left(\rho_{\text{A}}^{\text{U}}+\rho_{\text{B}}^{\text{L}}\right)\right)\right)}+\sqrt{\left(\left(2\left(c_{\text{B}}+c_{\text{t}}\right)+c_{\text{s\_}2\text{p}}\right)\left(\rho_{\text{A}}^{\text{U}}\left(1-\rho_{\text{B}}^{\text{L}}\right)/\left(\rho_{\text{A}}^{\text{U}}+\rho_{\text{B}}^{\text{L}}\right)\right)\right)}\right)}^{2}\left(\sigma_{y}^{2}/C\right)$
	else *ρ*_A_^L^, *ρ*_B_^U^	$2{\left(\sqrt{\left(\left(2\left(c_{\text{A}}+c_{\text{t}}\right)+c_{\text{s\_}2\text{p}}\right)\left(\left(\rho_{\text{B}}^{\text{U}}\left(1-\rho_{\text{A}}^{\text{L}}\right)\right)/\left(\rho_{\text{A}}^{\text{L}}+\rho_{\text{B}}^{\text{U}}\right)\right)\right)}+\sqrt{\left(\left(2\left(c_{\text{B}}+c_{\text{t}}\right)+c_{\text{s\_}2\text{p}}\right)\left(\rho_{\text{A}}^{\text{L}}\left(1-\rho_{\text{B}}^{\text{U}}\right)/\left(\rho_{\text{A}}^{\text{L}}+\rho_{\text{B}}^{\text{U}}\right)\right)\right)}\right)}^{2}\left(\sigma_{y}^{2}/C\right)$

*Note.σ*_*y*_^2^=*σ*_A_^2^+*σ*_B_^2^; *λ*=(2*c*_A_+2*c*_t_+*c*_s_2p_)/(2*c*_B_+2*c*_t_+*c*_s_2p_).

**Table 4:** Powers from the Monte Carlo simulations for maximin designs in the case of the treatment effect. For each pair of ranges of the intraclass correlations, the asymptotic efficiency of each design versus the most efficient design is given within brackets.

Treatment effect					
		*N*	Crossover design	Parallel design	Extended parallel design
*Type I error rate = 0.05*					
[*ρ*_A_^L^, *ρ*_A_^U^]	[*ρ*_B_^L^, *ρ*_B_^U^]				
[0.01,0.10]	[0.90,1.00]	30	0.857 (1)	0.648 (0.61)	**0.836** (0.96)
[0.01,0.10]	[0.90,1.00]	44	0.959 (1)	**0.822** (0.61)	0.950 (0.96)
[0.01,0.30]	[0.01,0.30]	36	0.916 (1)	0.805 (0.50)	**0.823** (0.76)
[0.01,0.30]	[0.70,1.00]	52	0.982 (1)	**0.819** (0.51)	0.922 (0.70)
[0.70,1.00]	[0.70,1.00]	10	**0.813** (1)	0.180 (0.15)	0.182 (0.15)
[0.90,1.00]	[0.90,1.00]	6	**0.902** (1)	0.088 (0.05)	0.087 (0.05)

*Type I error rate = 0.01*					
[*ρ*_A_^L^, *ρ*_A_^U^]	[*ρ*_B_^L^, *ρ*_B_^U^]				
[0.01,0.10]	[0.90,1.00]	45	0.854 (1)	0.618 (0.61)	**0.848** (0.96)
[0.01,0.10]	[0.90,1.00]	66	0.974 (1)	**0.829** (0.61)	0.967 (0.96)
[0.01,0.30]	[0.01,0.30]	55	0.931 (1)	0.817 (0.50)	**0.838** (0.76)
[0.01,0.30]	[0.70,1.00]	77	0.989 (1)	**0.822** (0.51)	0.931 (0.70)
[0.70,1.00]	[0.70,1.00]	16	**0.856** (1)	0.112 (0.15)	0.119 (0.15)
[0.90,1.00]	[0.90,1.00]	8	**0.849** (1)	0.029 (0.05)	0.030 (0.05)

*Note*. The power printed in bold indicates the design for which the sample calculation should yield a power of 80%. *N*: total sample size.

**Table 5:** Asymptotic variances of the ML estimator of the treatment effect and treatment by period interaction effect for different designs under heterogeneity of outcome variances.

Design	$\text{Var}\left({\hat{\beta }}_{\text{treat}}\right)$	$\text{Var}\left({\hat{\beta }}_{\text{time}\times \text{treat}}\right)$
Crossover design	((*ρ*_A_ − *ρ*_B_)^2^*p*(1 − *p*)+*ρ*_A_*ρ*_B_(1 − *ρ*_A_*ρ*_B_))/((*ρ*_A_+*ρ*_B_)(*ρ*_A_+*ρ*_B_+2*ρ*_A_*ρ*_B_))(*σ*_*y*_^2^/((1 − *p*)*pN*))	(1+2*ρ*_A_*ρ*_B_/(*ρ*_A_+*ρ*_B_))(*σ*_*y*_^2^/((1 − *p*)*pN*))

Parallel design	((*ρ*_B_/(*ρ*_A_+*ρ*_B_))(1/*p*)+(*ρ*_A_/(*ρ*_A_+*ρ*_B_))1/(1 − *p*))(*σ*_*y*_^2^/*N*)	na

Extended parallel design	((*ρ*_B_/(*ρ*_A_+*ρ*_B_))(1/*p*)+(*ρ*_A_/(*ρ*_A_+*ρ*_B_))(1/(1 − *p*))+((*ρ*_A_*ρ*_B_)/(*ρ*_A_+*ρ*_B_))(1/((1 − *p*)*p*)))(*σ*_*y*_^2^/(2*N*))	(((*ρ*_B_(1 − *ρ*_A_))/(*ρ*_A_+*ρ*_B_))(1/*p*)+((*ρ*_A_(1 − *ρ*_B_))/(*ρ*_A_+*ρ*_B_))(1/(1 − *p*)))(2*σ*_*y*_^2^/*N*)

*Note.σ*_*y*_^2^=*σ*_A_^2^+*σ*_B_^2^; na: not applicable.

**Table 6:** 

Treatment by period interaction				
		*N*	Crossover design	Extended parallel design
Type I error rate = 0.05				
[*ρ*_A_^L^, *ρ*_A_^U^]	[*ρ*_B_^L^, *ρ*_B_^U^]			
[0.01, 0.10]	[0.01, 0.10]	110	**0.799** (0.90)	0.840 (1)
[0.01, 0.30]	[0.01, 0.30]	130	**0.801** (0.76)	0.899 (1)
[0.70, 1.00]	[0.70, 1.00]	34	0.206 (0.15)	**0.830** (1)
[0.90, 1.00]	[0.90, 1.00]	15	0.111 (0.05)	**0.883** (1)

Type I error rate = 0.01				
[*ρ*_A_^L^, *ρ*_A_^U^]	[*ρ*_B_^L^, *ρ*_B_^U^]			
[0.01, 0.10]	[0.01, 0.10]	166	**0.808** (0.90)	0.848 (1)
[0.01, 0.30]	[0.01, 0.30]	194	**0.800** (0.76)	0.912 (1)
[0.70, 1.00]	[0.70, 1.00]	51	0.117 (0.15)	**0.840** (1)
[0.90, 1.00]	[0.90, 1.00]	23	0.048 (0.05)	**0.911** (1)

*Note*. The power printed in bold indicates the design for which the sample calculation should yield a power of 80%. *N*: total sample size.

## Data Availability

This study is not based on empirical data. However, the *R* programs that are used in this paper are available upon request from the corresponding author.

## Appendix

### A.1. Asymptotic Variance for the ML Estimator of the Treatment Effect

Let the vector of observed scores on the dependent variable for person *j*(=1,…, *N*) in a two-period design be denoted as $y_{j}=\left[\begin{array}{c} y_{1j}\\ y_{2j} \end{array}\right]$. The linear mixed model for the scores **y**_*j*_ can be expressed as follows:

(A.1) $$\begin{array}{c} y_{j}=X_{j}\beta +1u_{0j}+Q\varepsilon_{j}^{\text{A}}+\left(Ι-Q\right)\varepsilon_{j}^{\text{B}}, \end{array}$$

where **X**_*j*_ is the design matrix for subject *j*, **β** is the vector of regression coefficients, *u*_0*j*_ is the random person effect, $\varepsilon_{j}^{\text{A}}=\left[\begin{array}{c} \varepsilon_{1j}^{\text{A}}\\ \varepsilon_{2j}^{\text{A}} \end{array}\right]$ is the vector of residual scores under treatment A, and $\varepsilon_{j}^{\text{B}}=\left[\begin{array}{c} \varepsilon_{1j}^{\text{B}}\\ \varepsilon_{2j}^{\text{B}} \end{array}\right]$ is the vector of residual scores under treatment B. In (A.1), **1** = $\left[\begin{array}{c} 1\\ 1 \end{array}\right]$, **I** is the identity matrix, and **Q** is a matrix which indicates whether treatment A has been given or not in a particular period, so for a person *j* with treatment sequence AB, we have **Q** = $\left[\begin{array}{cc} 1 & 0\\ 0 & 0 \end{array}\right]$, indicating that A has been given in period 1 and treatment B in period 2.

Let **J** be a matrix with only ones (of order 2 by 2). The variance-covariance matrix of **y**_*j*_ can be derived as follows:

(A.2) $$\begin{array}{c} V_{j}=Q^{T}Q\sigma_{\varepsilon \text{A}}^{2}+{\left(I-Q\right)}^{T}\left(I-Q\right)\sigma_{\varepsilon \text{B}}^{2}+\sigma_{0}^{2}J. \end{array}$$

For the AA sequence, we have **Q** = **I**, and (A.2) can be rewritten as follows:

(A.3) $$\begin{array}{c} V_{j}=\sigma_{\varepsilon \text{A}}^{2}I+\sigma_{0}^{2}J. \end{array}$$

By applying the result that, for an *n* × *n* matrix, **X**=*a ***I**+*b ***J**, with *a* ≠ 0 and *a* ≠ −nb, the inverse is **X**^−1^=(1/*a*)(**I** − **J**(*b*/(nb+*a*))) ([18], p. 443), the inverse of the matrix in (A.3) can be written as follows:

(A.4) $$\begin{array}{c} V_{j}^{-1}=\frac{1}{\sigma_{\varepsilon \text{A}}^{2}}\left(I-J\left(\frac{\sigma_{0}^{2}}{2\sigma_{0}^{2}+\sigma_{\varepsilon \text{A}}^{2}}\right)\right)=\frac{1}{\sigma_{\varepsilon \text{A}}^{2}}\times \frac{1}{2\sigma_{0}^{2}+\sigma_{\varepsilon \text{A}}^{2}}\times \left(\begin{array}{cc} \sigma_{0}^{2}+\sigma_{\varepsilon \text{A}}^{2} & -\sigma_{0}^{2}\\ -\sigma_{0}^{2} & \sigma_{0}^{2}+\sigma_{\varepsilon \text{A}}^{2} \end{array}\right). \end{array}$$

For the *BB* sequence, we have **I − Q** = **I**, and in a similar way, we obtain

(A.5) $$\begin{array}{c} V_{j}^{-1}=\frac{1}{\sigma_{\varepsilon \text{B}}^{2}}\left(I-J\left(\frac{\sigma_{0}^{2}}{2\sigma_{0}^{2}+\sigma_{\varepsilon \text{B}}^{2}}\right)\right)=\frac{1}{\sigma_{\varepsilon \text{B}}^{2}}\times \frac{1}{2\sigma_{0}^{2}+\sigma_{\varepsilon \text{A}}^{2}}\times \left(\begin{array}{cc} \sigma_{0}^{2}+\sigma_{\varepsilon \text{B}}^{2} & -\sigma_{0}^{2}\\ -\sigma_{0}^{2} & \sigma_{0}^{2}+\sigma_{\varepsilon \text{B}}^{2} \end{array}\right). \end{array}$$

Finally, for the AB sequence and BA sequence, we can derive the following equations, respectively:

(A.6) $$\begin{array}{c} V_{j}^{-1}=\frac{1}{\left(\sigma_{0}^{2}+\sigma_{\varepsilon \text{A}}^{2}\right)\left(\sigma_{0}^{2}+\sigma_{\varepsilon \text{B}}^{2}\right)-\sigma_{0}^{4}}\times \left(\begin{array}{cc} \sigma_{0}^{2}+\sigma_{\varepsilon \text{B}}^{2} & -\sigma_{0}^{2}\\ -\sigma_{0}^{2} & \sigma_{0}^{2}+\sigma_{\varepsilon \text{A}}^{2} \end{array}\right),\\ V_{j}^{-1}=\frac{1}{\left(\sigma_{0}^{2}+\sigma_{\varepsilon \text{A}}^{2}\right)\left(\sigma_{0}^{2}+\sigma_{\varepsilon \text{B}}^{2}\right)-\sigma_{0}^{4}}\times \left(\begin{array}{cc} \sigma_{0}^{2}+\sigma_{\varepsilon \text{A}}^{2} & -\sigma_{0}^{2}\\ -\sigma_{0}^{2} & \sigma_{0}^{2}+\sigma_{\varepsilon \text{B}}^{2} \end{array}\right). \end{array}$$

The information matrix of the ML estimator of **β** can be written as

(A.7) Inf β^=∑j=1NXjTVj−1Xj.

Taking the inverse of the information matrix yields the asymptotic variance-covariance matrix of the ML estimators of **β**.

Different treatment sequences not only lead to different matrices **V**_*j*_ but also to different **X**_*j*_ matrices. As an example, we consider the crossover design. In total, we have *N* subjects, and *p* is the proportion of persons being allocated to the AB sequence. Let **β**^*T*^=(*β*_0_, *β*_1_, *β*_2_), where the regression coefficients represent the intercept, the treatment effect, and the time effect, respectively. For persons allocated to the AB sequence, we have $X_{j}=\left[\begin{array}{ccc} 1 & 0 & 0\\ 1 & 1 & 1 \end{array}\right]$, where *j*=1,…, Np. For persons allocated to the BA sequence, we have $X_{j}=\left[\begin{array}{ccc} 1 & 1 & 0\\ 1 & 0 & 1 \end{array}\right]$, where *j*=Np+1,…, *N*. We can now elaborate the matrix formulation in (A.7). For persons in the AB sequence, we have

(A.8) $$\begin{array}{c} X_{j}^{T}V_{j}^{-1}X_{j}=\frac{1}{\left(\sigma_{0}^{2}+\sigma_{\varepsilon \text{A}}^{2}\right)\sigma_{\varepsilon \text{B}}^{2}+\sigma_{0}^{2}\sigma_{\varepsilon \text{A}}^{2}}\left[\begin{array}{ccc} \sigma_{\varepsilon \text{A}}^{2}+\sigma_{\varepsilon \text{B}}^{2} & \sigma_{\varepsilon \text{A}}^{2} & \sigma_{\varepsilon \text{A}}^{2}\\ \sigma_{\varepsilon \text{A}}^{2} & \sigma_{0}^{2}+\sigma_{\varepsilon \text{A}}^{2} & \sigma_{0}^{2}+\sigma_{\varepsilon \text{A}}^{2}\\ \sigma_{\varepsilon \text{A}}^{2} & \sigma_{0}^{2}+\sigma_{\varepsilon \text{A}}^{2} & \sigma_{0}^{2}+\sigma_{\varepsilon \text{A}}^{2} \end{array}\right]. \end{array}$$

and for persons in the BA sequence, we obtain

(A.9) $$\begin{array}{c} X_{j}^{T}V_{j}^{-1}X_{j}=\frac{1}{\left(\sigma_{0}^{2}+\sigma_{\varepsilon \text{A}}^{2}\right)\sigma_{\varepsilon \text{B}}^{2}+\sigma_{0}^{2}\sigma_{\varepsilon \text{A}}^{2}}\left[\begin{array}{ccc} \sigma_{\varepsilon \text{A}}^{2}+\sigma_{\varepsilon \text{B}}^{2} & \sigma_{\varepsilon \text{A}}^{2} & \sigma_{\varepsilon \text{B}}^{2}\\ \sigma_{\varepsilon \text{A}}^{2} & \sigma_{0}^{2}+\sigma_{\varepsilon \text{A}}^{2} & -\sigma_{0}^{2}\\ \sigma_{\varepsilon \text{B}}^{2} & -\sigma_{0}^{2} & \sigma_{0}^{2}+\sigma_{\varepsilon \text{B}}^{2} \end{array}\right]. \end{array}$$

The information matrix in (A.7) can be obtained by summating the expressions in (A.8) and (A.9) across all persons in each of the treatment sequences, and the asymptotic variance-covariance matrix of the ML estimators then results by taking the inverse of this matrix. We are interested in the variance of the treatment effect estimator, which is the entry in row 2 and column 2 of the resulting variance-covariance matrix:

(A.10) $$\begin{array}{c} \text{Var}\left({\hat{\beta }}_{1}\;|\;\xi_{\text{crossover}}\right)=\frac{{\left(\sigma_{\varepsilon \text{B}}^{2}-\sigma_{\varepsilon \text{A}}^{2}\right)}^{2}p\left(1-p\right)+\left(\sigma_{0}^{2}+\sigma_{\varepsilon \text{A}}^{2}\right)\sigma_{\varepsilon \text{B}}^{2}+\sigma_{0}^{2}\cdot \sigma_{\varepsilon \text{A}}^{2}}{\left(4\sigma_{0}^{2}+\sigma_{\varepsilon \text{A}}^{2}+\sigma_{\varepsilon \text{B}}^{2}\right)p\left(1-p\right)N}, \end{array}$$

which, noting that *ρ*_A_=*σ*_0_^2^/(*σ*_0_^2^+*σ*_*ε*A_^2^), *ρ*_B_=*σ*_0_^2^/(*σ*_0_^2^+*σ*_*ε*B_^2^), and *σ*_*y*_^2^=2*σ*_0_^2^+*σ*_*ε*A_^2^+*σ*_*ε*B_^2^, can be written as the expression given in Table 5. Along similar lines, the variance of the treatment effect estimator for a parallel and an extended parallel design, as shown in Table 5, can be derived.

### A.2. Asymptotic Variance for the ML Estimator of the Treatment by Period Interaction Effect

The derivations of the variance of treatment by period interaction estimator are also similar, but start from another model and corresponding design matrices for the fixed regression coefficients. Now **β**^*T*^=(*β*_0_, *β*_1_, *β*_2_, *β*_3_), where the regression coefficients represent the intercept, the treatment effect, the time effect, and the treatment by period interaction effect, respectively. When considering again a crossover trial as an example, for persons assigned to the AB sequence, we have $X_{j}=\left[\begin{array}{cccc} 1 & 0 & 0 & 0\\ 1 & 1 & 1 & 1 \end{array}\right]$, where *j*=1,…, Np. For persons assigned to the BA sequence, we have $X_{j}=\left[\begin{array}{cccc} 1 & 1 & 0 & 0\\ 1 & 0 & 1 & 0 \end{array}\right]$, where *j*=Np+1,…, *N*. For persons in the AB sequence, we obtain

(A.11) $$\begin{array}{c} X_{j}^{T}V_{j}^{-1}X_{j}=\frac{1}{\left(\sigma_{0}^{2}+\sigma_{\varepsilon \text{A}}^{2}\right)\sigma_{\varepsilon \text{B}}^{2}+\sigma_{0}^{2}\sigma_{\varepsilon \text{A}}^{2}}\left[\begin{array}{cccc} \sigma_{\varepsilon \text{A}}^{2}+\sigma_{\varepsilon \text{B}}^{2} & \sigma_{\varepsilon \text{A}}^{2} & \sigma_{\varepsilon \text{A}}^{2} & \sigma_{\varepsilon \text{A}}^{2}\\ \sigma_{\varepsilon \text{A}}^{2} & \sigma_{0}^{2}+\sigma_{\varepsilon \text{A}}^{2} & \sigma_{0}^{2}+\sigma_{\varepsilon \text{A}}^{2} & \sigma_{0}^{2}+\sigma_{\varepsilon \text{A}}^{2}\\ \sigma_{\varepsilon \text{A}}^{2} & \sigma_{0}^{2}+\sigma_{\varepsilon \text{A}}^{2} & \sigma_{0}^{2}+\sigma_{\varepsilon \text{A}}^{2} & \sigma_{0}^{2}+\sigma_{\varepsilon \text{A}}^{2}\\ \sigma_{\varepsilon \text{A}}^{2} & \sigma_{0}^{2}+\sigma_{\varepsilon \text{A}}^{2} & \sigma_{0}^{2}+\sigma_{\varepsilon \text{A}}^{2} & \sigma_{0}^{2}+\sigma_{\varepsilon \text{A}}^{2} \end{array}\right], \end{array}$$

and for persons in the BA sequence, we obtain

(A.12) $$\begin{array}{c} X_{j}^{T}V_{j}^{-1}X_{j}=\frac{1}{\left(\sigma_{0}^{2}+\sigma_{\varepsilon \text{A}}^{2}\right)\sigma_{\varepsilon \text{B}}^{2}+\sigma_{0}^{2}\sigma_{\varepsilon \text{A}}^{2}}\left[\begin{array}{cccc} \sigma_{\varepsilon \text{A}}^{2}+\sigma_{\varepsilon \text{B}}^{2} & \sigma_{\varepsilon \text{A}}^{2} & \sigma_{\varepsilon \text{B}}^{2} & 0\\ \sigma_{\varepsilon \text{A}}^{2} & \sigma_{0}^{2}+\sigma_{\varepsilon \text{A}}^{2} & -\sigma_{0}^{2} & 0\\ \sigma_{\varepsilon \text{B}}^{2} & -\sigma_{0}^{2} & \sigma_{0}^{2}+\sigma_{\varepsilon \text{B}}^{2} & 0\\ \text{0} & 0 & 0 & 0 \end{array}\right]. \end{array}$$

The information matrix in (A.7) is obtained by summating the expressions in (A.11) and (A.12) across all persons in both treatment sequences, and the asymptotic variance-covariance matrix of the ML estimators then results by taking the inverse of this matrix. We are interested in the variance of the treatment by period interaction effect estimator, which is the entry in row 4 and column 4 of the variance-covariance matrix:

(A.13) $$\begin{array}{c} \text{Var}\left({\hat{\beta }}_{3}\;|\;\xi_{\text{cross}-\text{over}}\right)=\frac{\left(4\sigma_{0}^{2}+\sigma_{\varepsilon \text{A}}^{2}+\sigma_{\varepsilon \text{B}}^{2}\right)}{p\left(1-p\right)N}, \end{array}$$

which can be rewritten in terms of *ρ*_A_, *ρ*_B_, and *σ*_*y*_^2^, as the expression in Table 5. Along similar lines, the variance of the treatment by period interaction effect estimator for an extended parallel design, as shown in Table 5, can be derived.

### B. Derivation of Optimal Allocations under a Budget Constraint

The asymptotic variance of the treatment effect estimator is minimized as a function of *p*, the allocation proportion, given a fixed budget *C*. For a crossover, parallel, and extended parallel design, *p* is the proportion allocated to the sequence AB, A, and AA, respectively. The variance for a crossover design as given in Table 5 can be rewritten in terms of the research costs and budget *C*, employing the cost function in (5) and noting that *c*_sc_ = *c*_s_2p_:

(B.1) $$\begin{array}{c} \text{Var}\left({\hat{\beta }}_{1}\;|\;\xi_{\text{crossover}}\right)=\left(\frac{{\left(\rho_{\text{A}}-\rho_{\text{B}}\right)}^{2}p\left(1-p\right)+\rho_{\text{A}}\rho_{\text{B}}\left(1-\rho_{\text{A}}\rho_{\text{B}}\right)}{\left(\rho_{\text{A}}+\rho_{\text{B}}\right)\left(\rho_{\text{A}}+\rho_{\text{B}}+2\rho_{\text{A}}\rho_{\text{B}}\right)}\right)\left(\frac{\sigma_{y}^{2}}{\left(1-p\right)pC}\right)\left(c_{\text{A}}+c_{\text{B}}+2c_{\text{t}}+c_{\text{s\_}2\text{p}}\right)=\left({\left(\rho_{\text{A}}-\rho_{\text{B}}\right)}^{2}+\frac{\rho_{\text{A}}\rho_{\text{B}}\left(1-\rho_{\text{A}}\rho_{\text{B}}\right)}{\left(1-p\right)p}\right)\left(\frac{\sigma_{y}^{2}\left(c_{\text{A}}+c_{\text{B}}+2c_{\text{t}}+c_{\text{s\_}2\text{p}}\right)}{\left(\rho_{\text{A}}+\rho_{\text{B}}\right)\left(\rho_{\text{A}}+\rho_{\text{B}}+2\rho_{\text{A}}\rho_{\text{B}}\right)C}\right). \end{array}$$

It is easy to see that this expression is minimized if *p*(1 − *p*) is maximized, which is the case if *p*=0.5.

For a parallel design, the variance of the treatment effect estimator as given in Table 5 can be rewritten in terms of the cost function in (4) as follows:

(B.2) $$\begin{array}{c} \text{Var}\left({\hat{\beta }}_{1}\;|\;\xi_{\text{parallel}}\right)=\left(\left(\frac{\rho_{\text{B}}}{\rho_{\text{A}}+\rho_{\text{B}}}\right)\frac{1}{p}+\left(\frac{\rho_{\text{A}}}{\rho_{\text{A}}+\rho_{\text{B}}}\right)\frac{1}{\left(1-p\right)}\right)\frac{\sigma_{y}^{2}}{C}\left(c_{\text{sp}}+c_{\text{t}}+pc_{\text{A}}+\left(1-p\right)c_{\text{B}}\right). \end{array}$$

Taking the derivative of (B.2) with respect to *p* yields the following expression:

(B.3) $$\begin{array}{c} \left(\left(\left(2\left(c_{\text{sp}}+c_{\text{t}}\right)+c_{\text{A}}+c_{\text{B}}\right)\left(\frac{\rho_{\text{A}}}{\rho_{\text{A}}+\rho_{\text{B}}}\right)-\left(c_{\text{sp}}+c_{\text{t}}+c_{\text{B}}\right)\right)p^{2}+2\left(c_{\text{sp}}+c_{\text{t}}+c_{\text{B}}\right)\left(\frac{\rho_{\text{B}}}{\rho_{\text{A}}+\rho_{\text{B}}}\right)p-\left(c_{\text{sp}}+c_{\text{t}}+c_{\text{B}}\right)\left(\frac{\rho_{\text{B}}}{\rho_{\text{A}}+\rho_{\text{B}}}\right)\right)\frac{\sigma_{y}^{2}}{Cp^{2}{\left(1-p\right)}^{2}}. \end{array}$$

Solving the expression for *p* gives two solutions, one of which turns out to give a minimum (the second derivative of the variance as a function of *p* is positive for this particular value):

(B.4) $$\begin{array}{c} p=\frac{\sqrt{\left(c_{\text{sp}}+c_{\text{t}}+c_{\text{B}}\right)\rho_{\text{B}}}}{\sqrt{\left(c_{\text{sp}}+c_{\text{t}}+c_{\text{B}}\right)\rho_{\text{B}}}+\sqrt{\left(c_{\text{sp}}+c_{\text{t}}+c_{\text{A}}\right)\rho_{\text{A}}}}. \end{array}$$

Finally, for an extended parallel design, the variance of the treatment effect estimator as given in Table 5 can be rewritten in terms of the cost function in (6) noting that *c*_sep_ = *c*_s_2p_:

(B.5) $$\begin{array}{c} \text{Var}\left({\hat{\beta }}_{1}\;|\;\xi_{\text{extended parallel}}\right)=\left(\left(\frac{\rho_{\text{B}}}{\rho_{\text{A}}+\rho_{\text{B}}}\right)\frac{1}{p}+\left(\frac{\rho_{\text{A}}}{\rho_{\text{A}}+\rho_{\text{B}}}\right)\frac{1}{\left(1-p\right)}+\left(\frac{\rho_{\text{A}}\rho_{\text{B}}}{\rho_{\text{A}}+\rho_{\text{B}}}\right)\frac{1}{\left(1-p\right)p}\right)\frac{\sigma_{y}^{2}\left(c_{\text{s\_}2\text{p}}+2pc_{\text{A}}+2\left(1-p\right)c_{\text{B}}+2c_{\text{t}}\right)}{2C}. \end{array}$$

Taking the derivative with respect to *p* yields the following expression:

(B.6) $$\begin{array}{c} \left(\left(\left(c_{\text{s\_}2\text{p}}+2c_{\text{t}}\right)\left(\frac{\rho_{\text{A}}-\rho_{\text{B}}}{\rho_{\text{A}}+\rho_{\text{B}}}\right)-2c_{\text{B}}\left(\frac{\left(1+\rho_{\text{A}}\right)\rho_{\text{B}}}{\rho_{\text{A}}+\rho_{\text{B}}}\right)+2c_{\text{A}}\left(\frac{\left(1+\rho_{\text{B}}\right)\rho_{\text{A}}}{\rho_{\text{A}}+\rho_{\text{B}}}\right)\right)p^{2}+2\left(c_{\text{s\_}2\text{p}}+2c_{\text{t}}+2c_{\text{B}}\right)\left(\frac{\rho_{\text{B}}\left(1+\rho_{\text{A}}\right)}{\rho_{\text{A}}+\rho_{\text{B}}}\right)p-\left(c_{\text{s\_}2\text{p}}+2c_{\text{t}}+2c_{\text{B}}\right)\left(\frac{\rho_{\text{B}}\left(1+\rho_{\text{A}}\right)}{\rho_{\text{A}}+\rho_{\text{B}}}\right)\right)\times \frac{\sigma_{y}^{2}}{2Cp^{2}{\left(1-p\right)}^{2}}. \end{array}$$

Solving (B.6) for *p* gives two solutions, one of which turns out to give a minimum (the second derivative of the variance as a function of *p* is positive for his particular value):

(B.7) $$\begin{array}{c} p=\frac{\sqrt{\left(c_{\text{s\_}2\text{p}}+2\left(c_{\text{t}}+c_{\text{B}}\right)\right)\left(1+\rho_{\text{A}}\right)\rho_{\text{B}}}}{\sqrt{\left(c_{\text{s\_}2\text{p}}+2\left(c_{\text{t}}+c_{\text{B}}\right)\right)\left(1+\rho_{\text{A}}\right)\rho_{\text{B}}}+\sqrt{\left(c_{\text{s\_}2\text{p}}+2\left(c_{\text{t}}+c_{\text{A}}\right)\right)\left(1+\rho_{\text{B}}\right)\rho_{\text{A}}}}. \end{array}$$

Substituting the optimal allocation *p*=0.5, and the allocations in (B.4) and (B.7) into the corresponding expressions for the variance of the treatment effect estimator yields the optimal variances as given in Table 1 of the main text. Derivations along similar lines can be done if interest is in minimizing the variance of the treatment by period effect estimator. These result in the allocations and variances, as also presented in Table 1 of the main text.

### C. Derivation of Maximin Designs and Variances of the Effect Estimators

Maximin designs optimize the allocation to the treatment sequences under the worst case, that is, the maximum variance of the treatment effect estimator across plausible ranges of the model parameters, here the intraclass correlations *ρ*_A_ and *ρ*_B_.

For crossover designs, the variance of the treatment effect estimator under optimal allocation to the treatments is (see Table 1 of the main text)

(C.1) $$\begin{array}{c} \text{Var}\left({\hat{\beta }}_{1}\;|\;\xi_{\text{crossover}}\right)=\left(c_{\text{A}}+c_{\text{B}}+2c_{\text{t}}+c_{\text{s\_}2\text{p}}\right)\left(1-\frac{2\rho_{\text{A}}\rho_{\text{B}}}{\rho_{\text{A}}+\rho_{\text{B}}}\right)\frac{\sigma_{y}^{2}}{C}. \end{array}$$

By taking the derivatives with respect to *ρ*_A_ and *ρ*_B_, it can be shown that this expression decreases as a function of these parameters. This implies that the worst case occurs for the lower bounds of the ranges for *ρ*_A_ and *ρ*_B_. This yields the expression for the variance of the treatment effect estimator in the case of a maximin crossover design in Table 2 of the main text.

Similarly, for a parallel design, we have to examine for which values of *ρ*_A_ and *ρ*_B_ the following expression is maximized:

(C.2) $$\begin{array}{c} \text{Var}\left({\hat{\beta }}_{1}\;|\;\xi_{\text{parallel}}\right)=\left(\sqrt{\left(c_{\text{A}}+c_{\text{t}}+c_{\text{sp}}\right)\left(\frac{\rho_{\text{B}}}{\rho_{\text{A}}+\rho_{\text{B}}}\right)}\right.+{\left.\sqrt{\left(c_{\text{B}}+c_{\text{t}}+c_{\text{sp}}\right)\left(\frac{\rho_{\text{A}}}{\rho_{\text{A}}+\rho_{\text{B}}}\right)}\right)}^{2}\frac{\sigma_{y}^{2}}{C}. \end{array}$$

For derivations purposes, it is more convenient to rewrite (C.2) in terms of *ϕ*=*ρ*_B_/*ρ*_A_:

(C.3) $$\begin{array}{c} \text{Var}\left({\hat{\beta }}_{1}\;|\;\xi_{\text{parallel}}\right)=\left(\sqrt{\left(c_{\text{A}}+c_{\text{t}}+c_{\text{sp}}\right)\left(\frac{\phi }{1+\phi }\right)}\right.+{\left.\sqrt{\left(c_{\text{B}}+c_{\text{t}}+c_{\text{sp}}\right)\left(\frac{1}{1+\phi }\right)}\right)}^{2}\frac{\sigma_{y}^{2}}{C}. \end{array}$$

Taking the derivative of (C.3) with respect to *ϕ*, we find that the variance increases as a function of *ϕ* as long as *ϕ* ≤ (*c*_A_+*c*_t_+*c*_sp_)/(*c*_B_+*c*_t_+*c*_sp_) and decreases as a function of *ϕ* if *ϕ* > (*c*_A_+*c*_t_+*c*_sp_)/(*c*_B_+*c*_t_+*c*_sp_). So, if we can choose *ρ*_A_ and *ρ*_B_ from their plausible ranges such that *ρ*_B_/*ρ*_A_=(*c*_A_+*c*_t_+*c*_sp_)/(*c*_B_+*c*_t_+*c*_sp_), this maximizes (C.2). On the other hand, if even the lower bound of *ρ*_A_, *ρ*_A_^L^, and the upper bound of *ρ*_B_, *ρ*_B_^U^, yield *ρ*_B_^L^/*ρ*_A_^U^ < (*c*_A_+*c*_t_+*c*_sp_)/(*c*_B_+*c*_t_+*c*_sp_), then choose *ρ*_A_^L^ and *ρ*_B_^U^. Furthermore, if (*c*_A_+*c*_t_+*c*_sp_)/(*c*_B_+*c*_t_+*c*_sp_) < *ρ*_B_^L^/*ρ*_A_^U^, then choose *ρ*_A_^U^ and *ρ*_B_^L^. Substituting the maximin values of *ρ*_A_ and *ρ*_B_ into (C.2) will result in the variances of the treatment effect estimator as displayed in Table 2 of the main text.

For the extended parallel design, the variance for optimal allocation to the treatment sequences is given in Table 1 of the main text:

(C.4) $$\begin{array}{c} \text{Var}\left({\hat{\beta }}_{1}\;|\;\xi_{\text{extended parallel}}\right)={\left(\sqrt{\left(2\left(c_{\text{A}}+c_{\text{t}}\right)+c_{\text{s\_}2\text{p}}\right)\left(\frac{\rho_{\text{B}}\left(1+\rho_{\text{A}}\right)}{\rho_{\text{A}}+\rho_{\text{B}}}\right)}+\sqrt{\left(2\left(c_{\text{B}}+c_{\text{t}}\right)+c_{\text{s\_}2\text{p}}\right)\left(\frac{\rho_{\text{A}}\left(1+\rho_{\text{B}}\right)}{\rho_{\text{A}}+\rho_{\text{B}}}\right)}\right)}^{2}\frac{\sigma_{y}^{2}}{2C}. \end{array}$$

Taking the derivative of (C.4) with respect to *ρ*_A_ shows that the variance of the treatment effect estimator increases as a function of *ρ*_A_ as long as *λ*(1 − *ρ*_B_)^2^/(*ρ*_B_(1+*ρ*_B_)) ≤ (1+*ρ*_A_)/*ρ*_A_ where *λ*=(2(*c*_A_+*c*_t_)+*c*_s_2p_)/(2(*c*_B_+*c*_t_)+*c*_s_2p_) and decreases if *λ*(1 − *ρ*_B_)^2^/(*ρ*_B_(1+*ρ*_B_)) > (1+*ρ*_A_)/*ρ*_A_. Taking into account a feasible range for *ρ*_A_, the value for *ρ*_A_ maximizing the variance in (C.4), *ρ*_A_^*∗*^, therefore is

(C.5) $$\begin{array}{c} \rho_{\text{A}}^{*}=\frac{\rho_{\text{B}}\left(1+\rho_{\text{B}}\right)}{\lambda {\left(1-\rho_{\text{B}}\right)}^{2}-\rho_{\text{B}}\left(1+\rho_{\text{B}}\right)}\;\text{if}\;\rho_{\text{A}}^{\text{L}}\leq \rho_{\text{A}}^{*}\leq \rho_{\text{A}}^{\text{U}}\;\text{or}\;\frac{1}{\rho_{\text{A}}^{\text{U}}}+1\leq \frac{\lambda {\left(1-\rho_{\text{B}}\right)}^{2}}{\left(1+\rho_{\text{B}}\right)\rho_{\text{B}}}\leq \frac{1}{\rho_{\text{A}}^{\text{L}}}+1, \end{array}$$

(C.6) $$\begin{array}{c} \rho_{\text{A}}^{*}=\rho_{\text{A}}^{\text{U}}\;\text{if}\;\rho_{\text{A}}^{\text{U}}\leq \rho_{\text{A}}^{*}\;\text{or}\;\frac{\lambda {\left(1-\rho_{\text{B}}\right)}^{2}}{\left(1+\rho_{\text{B}}\right)\rho_{\text{B}}}<\frac{1}{\rho_{\text{A}}^{\text{U}}}+1, \end{array}$$

(C.7) $$\begin{array}{c} \rho_{\text{A}}^{*}=\rho_{\text{A}}^{\text{L}}\;\text{if}\;\rho_{\text{A}}^{*}<\rho_{\text{A}}^{\text{L}}\;\text{or}\;\frac{1}{\rho_{\text{A}}^{\text{L}}}+1<\frac{\lambda {\left(1-\rho_{\text{B}}\right)}^{2}}{\left(1+\rho_{\text{B}}\right)\rho_{\text{B}}}. \end{array}$$

Taking the derivative of (C.4) with respect to *ρ*_B_ shows that the variance increases as a function of *ρ*_B_ as long as (1 − *ρ*_A_)^2^/(*λρ*_A_(1+*ρ*_A_)) ≤ (1+*ρ*_B_)/*ρ*_B_ and decreases if (1 − *ρ*_A_)^2^/(*λρ*_A_(1+*ρ*_A_)) > (1+*ρ*_B_)/*ρ*_B_. So, also taking into account a feasible range for *ρ*_B_, we have as maximin value for *ρ*_B_:

(C.8) $$\begin{array}{c} \rho_{\text{B}}^{*}=\frac{\lambda \rho_{\text{A}}\left(1+\rho_{\text{A}}\right)}{{\left(1-\rho_{\text{A}}\right)}^{2}-\lambda \rho_{\text{A}}\left(1+\rho_{\text{A}}\right)}\;\text{if}\;\rho_{\text{B}}^{\text{L}}\leq \rho_{\text{B}}^{*}\leq \rho_{\text{B}}^{\text{U}}\;\text{or}\;\frac{1}{\rho_{\text{B}}^{\text{U}}}+1\leq \frac{{\left(1-\rho_{\text{A}}\right)}^{2}}{\lambda \left(1+\rho_{\text{A}}\right)\rho_{\text{A}}}\leq \frac{1}{\rho_{\text{B}}^{\text{L}}}+1, \end{array}$$

(C.9) $$\begin{array}{c} \rho_{\text{B}}^{*}=\rho_{\text{B}}^{\text{U}}\;\text{if}\;\rho_{\text{B}}^{\text{U}}<\rho_{\text{B}}^{*}\;\text{or}\;\frac{{\left(1-\rho_{\text{A}}\right)}^{2}}{\lambda \left(1+\rho_{\text{A}}\right)\rho_{\text{A}}}<\frac{1}{\rho_{\text{B}}^{\text{U}}}+1, \end{array}$$

(C.10) $$\begin{array}{c} \rho_{\text{B}}^{*}=\rho_{\text{B}}^{\text{L}}\;\text{if}\;\rho_{\text{B}}^{*}<\rho_{\text{B}}^{\text{L}}\;\text{or}\;\frac{1}{\rho_{\text{B}}^{\text{L}}}+1<\frac{{\left(1-\rho_{\text{A}}\right)}^{2}}{\lambda \left(1+\rho_{\text{A}}\right)\rho_{\text{A}}}. \end{array}$$

For each of the intraclass correlations, *ρ*_A_ and *ρ*_B_, there are three possible values that maximize the variance of the treatment effect estimator. Not all values can cooccur. (C.5) and (C.8) imply that (1+*ρ*_B_^*∗*^)^2^(1+*ρ*_A_^*∗*^)^2^=(1−*ρ*_B_^*∗*^)^2^(1−*ρ*_A_^*∗*^)^2^, which in turn is true only if *ρ*_A_^*∗*^=*ρ*_B_^*∗*^=0, an unrealistic condition that we will not consider. Furthermore, if *ρ*_B_^*∗*^=*ρ*_B_^L^ then (1/*ρ*_B_^L^)+1 < ((1 − *ρ*_A_)^2^)/(*λ*(1+*ρ*_A_)*ρ*_A_)(C.10), which implies that *λ* < (((1 − *ρ*_A_)^2^)/((1+*ρ*_A_)*ρ*_A_)) × (*ρ*_B_^L^/(*ρ*_B_^L^+1)). If at the same time *ρ*_A_^*∗*^=*ρ*_B_(1+*ρ*_B_)/(*λ*(1 − *ρ*_B_)^2^ − *ρ*_B_(1+*ρ*_B_)), then also *λ*=((1+*ρ*_A_)/*ρ*_A_) × (*ρ*_B_^L^(*ρ*_B_^L^+1)/(1 − *ρ*_B_^L^)^2^). The latter two conditions for *λ* imply that (1+*ρ*_A_)^2^(1+*ρ*_B_^L^)^2^ < (1 − *ρ*_A_)^2^(1 − *ρ*_B_^L^)^2^, which is never true. On the other hand, if *ρ*_A_^*∗*^=*ρ*_B_(1+*ρ*_B_)/(*λ*(1 − *ρ*_B_)^2^ − *ρ*_B_(1+*ρ*_B_)), then it is possible that *ρ*_B_^*∗*^=*ρ*_B_^U^. It can also be shown that if *ρ*_B_^*∗*^=*λρ*_A_(1+*ρ*_A_)/((1 − *ρ*_A_)^2^ − *λρ*_A_(1+*ρ*_A_)), then it is only possible that *ρ*_A_^*∗*^=*ρ*_A_^U^. So, all this implies that we can rewrite the “if” conditions in (C.5)–(C.7), with *ρ*_B_ replaced by *ρ*_B_^U^, and the “if” conditions in (C.8)–(C.10), with *ρ*_A_ replaced by *ρ*_A_^U^.

Finally, we can show that the combination *ρ*_A_^*∗*^=*ρ*_A_^L^ and *ρ*_B_^*∗*^=*ρ*_B_^L^ cannot occur. This implies the inequalities in (C.7) and (C.10), which in turn imply that (1/*ρ*_A_^L^)+1 < (*λ*(1 − *ρ*_B_^U^)^2^)/((1+*ρ*_B_^U^)*ρ*_B_^U^) and (1/*ρ*_B_^L^)+1 < ((1 − *ρ*_A_^U^)^2^)/(*λ*(1+*ρ*_A_^U^)*ρ*_A_^U^), respectively, which imply *λ* > ((1+*ρ*_A_^L^)/*ρ*_A_^L^) × ((1+*ρ*_B_^U^)*ρ*_B_^U^/(1 − *ρ*_B_^U^)^2^) and *λ* < ((1 − *ρ*_A_^U^)^2^/((1+*ρ*_A_^U^)*ρ*_A_^U^)) × (*ρ*_B_^L^/(*ρ*_B_^L^+1)), respectively, which are incompatible. All other combinations of the maximin values *ρ*_A_^*∗*^ and *ρ*_B_^*∗*^ can be shown to be possible. These results lead to the procedure for determining the maximin design as delineated in Table 2 of the main text for an extended parallel design.

Similar derivations can be given for the maximin versions of a crossover and extended parallel design in the case interest is in the treatment by period interaction effect. These derivations are, upon request, available from the author.

### D. Powers from the Monte Carlo Simulations for Maximin Designs in the Case of the Treatment by Period Interaction

Table 6 provides the simulated powers for maximin designs in the case of the treatment by period interaction. For each pair of ranges of the intraclass correlations, the asymptotic efficiency of each design versus the most efficient design is given within brackets.
